# Genetic Elucidation of Quorum Sensing and Cobamide Biosynthesis in Divergent Bacterial-Fungal Associations Across the Soil-Mangrove Root Interface

**DOI:** 10.3389/fmicb.2021.698385

**Published:** 2021-10-05

**Authors:** Zhengyuan Zhou, Ruiwen Hu, Yanmei Ni, Wei Zhuang, Zhiwen Luo, Weiming Huang, Qingyun Yan, Zhili He, Qiuping Zhong, Cheng Wang

**Affiliations:** ^1^Environmental Microbiomics Research Center, School of Environmental Science and Engineering, Southern Marine Science and Engineering Guangdong Laboratory (Zhuhai), Sun Yat-sen University, Guangzhou, China; ^2^Guangdong Agribusiness Tropical Agriculture Institute, Guangzhou, China

**Keywords:** network, bacterial-fungal association, endosphere, quorum sensing, cobamide

## Abstract

Plant roots in soil host a repertoire of bacteria and fungi, whose ecological interactions could improve their functions and plant performance. However, the potential microbial interactions and underlying mechanisms remain largely unknown across the soil-mangrove root interface. We herein analyzed microbial intra- and inter-domain network topologies, keystone taxa, and interaction-related genes across four compartments (non-rhizosphere, rhizosphere, episphere, and endosphere) from a soil-mangrove root continuum, using amplicon and metagenome sequencing technologies. We found that both intra- and inter-domain networks displayed notable differences in the structure and topology across four compartments. Compared to three peripheral compartments, the endosphere was a distinctive compartment harboring more dense co-occurrences with a higher average connectivity in bacterial-fungal network (2.986) than in bacterial (2.628) or fungal network (2.419), which could be related to three bacterial keystone taxa (*Vibrio*, *Anaerolineae*, and *Desulfarculaceae*) detected in the endosphere as they are known to intensify inter-domain associations with fungi and stimulate biofilm formation. In support of this finding, we also found that the genes involved in cell-cell communications by quorum sensing (*rhlI*, *lasI*, *pqsH*, and *lasR*) and aerobic cobamide biosynthesis (*cobG*, *cobF*, and *cobA*) were highly enriched in the endosphere, whereas anaerobic cobamide biosynthesis (encoded by *cbiT* and *cbiE*) was dominant in three peripheral compartments. Our results provide genetic evidence for the intensified bacterial-fungal associations of root endophytes, highlighting the critical role of the soil-root interface in structuring the microbial inter-domain associations.

## Introduction

The roots of soil-grown plants host diverse microbial communities, and microbe-microbe interactions (e.g., inhibition, facilitation, and competition) are highly prevalent and have emerged as an important feature of plant root ecosystems (Gomes et al., 2011; Duran et al., 2018). Recent studies have shown that intra- or inter-domain microbial interactions could sustain the harmony of biogeochemical processes and keep the nutritional status and ecological balance for plant health (Thatoi et al., 2013; Ma et al., 2016). One typical example is the association between arbuscular mycorrhizal fungi and nitrogen-fixing bacteria, and their synergistical interactions can result in better nutrient uptake and higher plant productivity compared to plants symbiotic with either of them alone (Banerjee et al., 2019). At the community level, inter-domain microbial interactions were specifically enriched with negative correlations between bacteria and filamentous eukaryotes in *Arabidopsis thaliana* roots, where bacterial communities were essential for plant survival and protection against filamentous eukaryotes (Zhang et al., 2019). These studies contribute to the growing body of evidence that the plant growth and health are closely connected to microbial interactions from soil-root system (Duran et al., 2018), which carry out key functions like synergistic effects on plant growth, maintenance of host-microbiota balance, and protection against environmental pathogens (Duran et al., 2018).

Current studies suggested that the soil-root microhabitat could be divided into four continuous compartments: non-rhizosphere (buck soil), rhizosphere (Saleem et al., 2018), episphere, and endosphere (Duran et al., 2018). Microbial communities in each compartment across the soil-root interface were shown to exhibit unique characteristics (Edwards et al., 2015; Duran et al., 2018). The rhizosphere could selectively gather specific bacterial and fungal populations *via* root exudates, which conversely exerted a marginal influence on microbes in the non-rhizosphere soil (Bais et al., 2006; Sasse et al., 2018). Compared to the rhizosphere, the episphere played a more critical role for the controlled entry of specific soil-borne microbes into the plant root, leading to the selective enrichment of Proteobacteria and the absence of soil-derived fungi in the endosphere (Ofek-Lalzar et al., 2014; Duran et al., 2018). In fact, the significant variations that bacterial and fungal communities underwent across the soil-root interface were irrespective of plant hosts, and microbial diversity inside plant roots was much lower than that in other three exterior root compartments (Edwards et al., 2015). These findings have greatly advanced our understanding of bacterial and fungal community structure and assembly in soil-root system. However, the intra- and inter-domain interactions of microbial communities, and their dynamics across the soil-root interface are poorly understood.

In order to unravel the organization and strength of microbial interactions, network analysis has been widely used as a promising tool to disentangle microbial co-occurrence in various environments, such as groundwater, oceans and soil environments (Shi et al., 2016). Recently, it has been applied to evaluate the microbe-microbe associations in plant roots (Chen et al., 2019; Zhang et al., 2020) and their responses to environmental parameters (Morriën et al., 2017; de Vries et al., 2018). More importantly, the topology-based network analysis provides an opportunity to identify the keystone taxa, which are strongly interconnected and have an important effect on communities (Agler et al., 2016; Ma et al., 2016). In despite of numerically inconspicuous, keystone taxa confer higher biotic connectivity to the microbial community and therefore can be predictors of community shifts and compositional turnover (Herren and McMahon, 2018). However, the profile of keystone taxa across the soil-root interface and how it relates with the microbial interactions are poorly understood.

Many secondary metabolites are usually bioactive and can perform key functions in microbial interactions. A well-established mechanism of cell-to-cell communications in plant roots is quorum sensing, which is defined to be a stimuli-response system related to cellular density (Miller and Bassler, 2001; Shi et al., 2016; Kareb and Aider, 2020). Early studies have shown that quorum sensing can regulate bioluminescence and biofilm formation by releasing a specific acylated homoserine lactone (HSL) signaling molecule, which is known as an autoinducer produced by *LuxI*-like proteins (Miller and Bassler, 2001), and distinct microbes can produce the same type of signaling molecules, which have a role in both inter- and intra-domain interactions (Banerjee et al., 2018). Similarly, cobamides are recognized as mediators of microbial interactions (Bertrand et al., 2015; Shelton et al., 2019; Olga et al., 2020), making contributions to the nucleotide biosynthesis and catabolism of carbon sources (Olga et al., 2020). It is important to note that, microbes of all domains need cobamides, but many of them rely on the surrounding species to complement. This facilitates the ubiquitous establishment of a network of cobamide-dependent interactions in coastal ecosystems (Olga et al., 2020). Many previous studies have investigated the profile of genes associated with quorum sensing and cobamide biosynthesis in the complex microbial communities; however, little is known about whether and how these genes are connected to the microbe-microbe interaction dynamics at a community level.

As an ecologically important coastal ecosystem, mangrove plays a critical role in carbon storage, climate mitigation, shoreline protection and nutrient filtering (Alongi, 2014). Increasing evidences have revealed a high diversity of bacterial and fungal communities inhabiting across the soil-mangrove root interface (Zhuang et al., 2020), and determined their importance for mangrove growth and function (Reef et al., 2010; Thatoi et al., 2013; Xie et al., 2014). For instance, biological nitrogen fixation performed by diazotrophic bacteria in the vicinity of mangrove roots accounted for 40–60% of the total nitrogen required by mangroves (Holguin et al., 2001; Reef et al., 2010). Rhizosphere fungi not only displayed ligninolytic, cellulolytic, and amylolytic activity (Thatoi et al., 2013), but also could help mangroves adapt to the waterlogged and nutrient-restricted environments (Xie et al., 2014). Despite the improved understanding of functional capabilities of root–associated bacterial and fungal communities, our knowledge of potential interactions and underlying mechanisms within microbial communities from soil-mangrove root system remains largely unknown.

In this study, we profiled bacterial and fungal communities across four compartments (non-rhizosphere, rhizosphere, episphere, and endosphere) of mangrove roots with the aim of inferring the structure of microbial intra- and inter-domain co-occurrence networks and exploring putative keystone taxa across the soil-root interface. Amplicon and metagenome sequencing were performed to examine network topology and profiles of quorum sensing genes, cobamide biosynthesis genes and microbes in microbial communities across the soil-mangrove root interface. This study uncovers how diverse taxonomic groups of bacteria and fungi can form metacommunity-scale networks of putative microbial intra- and inter-domain interactions, providing a basis for understanding complex spatial processes of soil-microbe-plant systems and engineering complex microbial consortia with predictable behaviors and robust outcomes.

## Materials and Methods

### Site Selection, Sampling, and Environmental Properties

The location of the sampling site is at the Shuidong Bay of Maoming City (21°30′38.82″ N, 111°0′37.27″E), Guangdong, China. In April 2019, we collected 12 individual mangrove saplings in a mangrove forest consisting of *Kandelia obovata* and *Sonneratia apetala*, and divided the samples from soil to root into four compartments: non-rhizosphere soil (Non), rhizosphere soil (Rhi), root episphere (Epi), and root endosphere (Endo). These compartments were prepared as described by Edwards et al. (2015) and Duran et al. (2018). The non-rhizosphere soil was shaken off the root, whereas the rhizosphere soil was the part of the soil at ∼1 mm thickness around the root and was washed with sterile water. The clean roots were subsequently washed three times to remove the remaining soil and placed into 1 × TE buffer supplemented with 0.1% Triton X-100 in a 50 mL Falcon tube. Next, we collected the episphere samples by washing and extensive shaking in 1 × TE buffer supplemented with 0.1% Triton X-100. The microbial biomass of episphere was obtained *via* filtering the resulting suspension through 0.22 μM pore size membranes (Nuclepore, Whatman, Meterstone, United Kingdom). To capture the endosphere microbial biomass, the roots were surface-sterilized for 1 min in 80% ethanol and then sterilized again for 1 min in 0.25% NaClO. Samples from all four compartments in the soil-root system were stored at −80°C until DNA extraction. For soils in the non-rhizosphere, pH, salinity (permillage), oxidation reduction potential (mV), moisture content (%), total carbon (mg/Kg), total nitrogen (mg/Kg), ammonium-N (mg/Kg), nitrate-N (mg/Kg), and nitrite-N (mg/Kg) were measured (Supplementary Table 1) by previously described (Li et al., 2019).

### DNA Extraction, PCR Amplification and Sequencing

For each of 12 mangrove saplings, approximately 0.5 g of non-rhizosphere and rhizosphere soil was used for DNA extraction using a Power Soil DNA Isolation Kit following the protocol provided by the manufacturer (MoBio, Carlsbad, CA, United States) with the modified sodium dodecyl sulfate extraction method (Zhou et al., 1996). The episphere compartment DNA was extracted using a Power Water DNA Isolation Kit following the protocol provided by the manufacturer (MoBio, Carlsbad, CA, United States). For the endosphere samples, DNA was extracted from plant samples with the Power Plant DNA Isolation Kit, according to the manufacturer’s protocol (Mo Bio Laboratories, Inc., Carlsbad, CA, United States) after thorough grinding with liquid nitrogen. The DNA quality of four compartments in the soil-root system was assessed by Nano Drop ND-2000 Spectrophotometer (Thermo Fisher Scientific, MA, United States) based on 260/280 and 260/230 nm ratios (Tu et al., 2016). Qualified DNA samples were diluted to 2 ng/μL for subsequent PCR amplification.

The V3-V4 region of 16S rRNA genes was amplified with the primer pair (forward primer, 5′- ACTCCTACGG GAGGCAGCA-3′; reverse primer, 5′- GGACTACHVGGGTW TCTAAT-3′) (Peng et al., 2017) and the ITS1 region of fungal ITS genes were amplified with the primer pair (forward primer, 5′-CTTGGTCATTTAGAGGAAGTAA-3′; reverse primer, 5′-G CTGCGTTCTTCATCGATGC-3′) (Blaalid et al., 2013). PCR amplification was conducted in a total volume of 50 μL containing 10 μL buffer, 0.2 μL Q5 high-fidelity DNA polymerase, 10 μL high GC enhancer, 1 μL dNTP, 10 μM of each primer and 60 ng microbial community DNA. Thermal cycling conditions were as follows: an initial denaturation at 95°C for 5 min, followed by 15 cycles at 95°C for 1 min, 50°C for 1 min and 72°C for 1 min, with a final extension at 72°C for 7 min. The PCR products from the first step PCR were purified with VAHTSTM DNA Clean Beads, and the second round PCR was performed in a 40 μL reaction containing 20 μL 2 × Phusion HF MM, 8 μL ddH_2_O, 10 μM of each primer and 10 μL PCR products from the first step. Thermal cycling conditions were as follows: an initial denaturation at 98°C for 30 s, followed by 10 cycles at 98°C for 10 s, 65°C for 30 s min and 72°C for 30 s, with a final extension at 72°C for 5 min. All PCR products were quantified by Quant-iT^TM^ dsDNA HS Reagent and pooled together. High-throughput sequencing analysis of bacterial rRNA genes and fungal ITS genes was performed on the purified, pooled samples using the Illumina Hiseq 2500 platform (2 × 250 paired ends) at Biomarker Technologies Corporation, Beijing, China.

### Sequence Analysis of 16S rRNA and ITS Gene Amplicons

Raw sequences were trimmed using Trimmomatic (Marc et al., 2012) and FLASH (Tanja and Salzberg, 2011), with a moving window of 50-bp and the quality threshold score of 30. After the singleton elimination, paired 16S rRNA amplicon sequences were then clustered into operational taxonomic units (OTUs) by UPARSE (Edgar, 2013) based on a 97% sequence identity using QIIME’s (QIIME: Quantitative Insights into Microbial Ecology) (Caporaso et al., 2010) open reference OTU picking strategy with the Greengenes 16S rRNA database (v.13.5) as a reference (DeSantis et al., 2006). Bacterial OTU was represented as OTUB, and fungal OTU was represented as OTUF. We eliminated the sequences matching “Chloroplast” and “Mitochondria” from the datasets. ITS sequences were processed by ITSx (Bengtsson-Palme et al., 2013) and clustered at a 97% sequence identity by UPARSE (Edgar, 2013). Fungal OTUs were checked for chimeric sequences using the Uchime reference against a dedicated chimera detection database (Nilsson et al., 2015), which was based on the UNITE database for fungi identification. On average, we obtained 70,914 and 77,186 high-quality 16S rRNA and ITS gene amplicon sequences per sample, respectively. For all samples, the 16S rRNA and ITS sequences were clustered into 1,290 and 1,240 OTUs (>0.01% of total abundance), respectively. The nucleotide sequences were deposited in SRA database under accession numbers PRJNA685020 and PRJNA685297.

### Network Construction and Analysis

Microbial intra- and inter-domain networks were constructed based on bacterial and fungal OTU relative abundances across each root compartment. Covariations were measured across 12 biological replicates to create each network. Only OTUs detected in 8 out of 12 replicate samples were used for network construction. Random matrix theory (RMT) was used to automatically identify the appropriate similarity threshold (St) before network construction, since RMT could minimized the uncertainty in network construction by using mathematically defined non-arbitrary correlation cut-off (Yuan et al., 2021). St defined the minimal strength of the connections between each pair of nodes (Zhou et al., 2010). Global network properties were characterized as described in Deng et al. (2012). All analyses were performed with the Molecular Ecological Network Analyses (MENA) Pipeline^1^ (Deng et al., 2012) and the network structures were visualized using Cytoscape 3.6.1 (Shannon et al., 2003) and Gephi 0.9.2-beta (Bastian et al., 2009).

### Module Detection and Node Role Identification

We examined network modularity for each network constructed in this study. A module is a set of nodes (OTUs) that are highly connected inside the group but few connected outside the group (Newman, 2006). Modules were identified using the greedy modularity optimization method in this study. Modularity (*M*) is an indicator that measures the extent of network module division, and *M* > 0.4 is used as the threshold to define module structure (Newman, 2006). Additionally, the connectivity of each node was determined according to its within-module connectivity (*Zi*) and among-module connectivity (*Pi)* (Roger, 2005). Based on the topological roles in the network, we organized node topology into four categories: network hubs (nodes with high connectivity in the entire network, *Zi* > 2.5 and *Pi* > 0.62), module hubs (nodes with high connectivity inside modules, *Zi* > 2.5), connectors (nodes that connect modules, *Pi* > 0.62) and peripherals (nodes with few outside connections, *Zi* < 2.5 and *Pi* < 0.62) (Olesen et al., 2007; Deng et al., 2012).

### Shotgun Metagenome Sequencing and Data Analysis

Metagenomic sequencing library preparation was performed as previously described (Zhuang et al., 2020). Briefly, using 1 microgram DNA with Illumina (NEB, United States) NEBNext^®^ Ultra^TM^ DNA Library Prep Kit as recommended by the manufacturer. The index codes were added to attribute sequence of each compartment sample, and these samples then were purified (AMPure XP system). Agilent 2100 bioanalyzer (Agilent Technologies, CA, United States) was used to check the libraries. Paired-end reads with poor quality (quality score ≤ 38; base N > 10 bp, and the overlap length between adapter and reads was greater than 15 bp) are filtered. The filtered clean reads (about 11.8–12.5 Gb per sample) were used for metagenomic analyses. The metagenomic assembly was performed using Megahit (Li et al., 2015) in default mode. MetaGeneMark (v 2.10) were employed to predict genes from the assembled contigs, and redundancy was removed using CD-HIT Software (Fu et al., 2012).

For the assembled metagenomes, MetaGeneMark v. 2.10 was used to predict open reading frames, CD-HIT v. 4.5.8 was used to construct Unigenes (Fu et al., 2012), and SoapAligner v. 2.21 was used for quality control. We used DIAMOND combined with the KEGG database (blastp, *e*-value ≤ 1e−5) (Moriya et al., 2007) to perform functional annotation on genes related to quorum sensing and cobamide biosynthesis. In detail, we first determined the processes of quorums sensing and cobamide biosynthesis by referring to relevant studies (Warren et al., 2002; Ng and Bassler, 2009; Mukherjee and Bassler, 2019; Shelton et al., 2019), and the quorum sensing and cobamide biosynthesis pathways in KEGG database. Key genes that were involved in the quorum sensing and cobamide biosynthesis pathways were subsequently identified. The keywords of each gene were determined by its gene code and corresponding protein name, and were further refined by manually checking the description (Supplementary Table 2). The gene reading counts were normalized to transcripts per-million (TPM) counts. Statistical enrichment of a given gene between compartments was determined by pairwise comparisons using two-tailed Fisher’s exact test, with confidence intervals at 99% significance and Benjamini–Hochberg correction (*P* < 0.05). The Mantel tests between microbial communities and environmental factors were performed in R with the mantel function in the package {ggcor}. To get the taxonomical profile of cobamide biosynthesis microbiomes, the sequences were extracted by the *seqtk* program (Shen et al., 2016), and subsequently annotated with the Kraken2 program (Wood et al., 2019). The metagenome sequencing data were deposited in SRA database with accession numbers of PRJNA613873.

### Root Exudate Analysis and Biofilm Microscopic Analysis

To identify potential substances related to biofilm formation, we used liquid chromatography–mass spectrometry (LC/MS) analysis (1290 infinity series UHPLC system, Agilent Technologies) to detect the root exudates. In detail, mangrove saplings were transferred to 1-L hydroponic tubes with sterile Milli-Q water, and hydroponic tubes were incubated at 24°C for 24 h for root exudates collection. Ethyl acetate and dichloromethane were used to extract the resulting solution in a rotary evaporator, and we subsequently frozen it. Next, the sample was dissolved in 100 μL methanol, and we added 400 μL extract solution (acetonitrile:methanol = 1:1) containing the internal standard (L-2-Chlorophenylalanine, 2 μg/mL). After vortexing for 30 s, the sample was sonicated with an ice-water bath and then incubated at −40°C. After the sample was centrifuged at 4°C, the supernatant was dried in a vacuum concentrator at 37°C and reconstituted in 200 μL acetonitrile (50%) via sonication on ice. The resulting solution was then centrifuged at 4°C, and 75 μL supernatant was finally transferred into a sterile glass vial for LC/MS analysis. We used positive and negative modes with ion spray voltage floating (ISVF) at 5,000 or −4,000 V, respectively. MS raw data files were transformed into the mzXML format by ProteoWizard, and subsequently processed via the R package XCMs (v3.2), including peak deconvolution, alignment, and integration.

To visualize mangrove root structure and biofilm formation, we used a hybrid microscope (REVOLVE 3, Echo Laboratories) to investigate slices of fresh roots in both brightfield and fluorescence field (488 nm, Mercury Free LED). Before microscopic examination, fresh mangrove roots stored in −80°C were washed by 80% ethanol for surface sterilization, and cut into slices using sterile blades. Afterward, the root slices were fixed with glutaraldehyde (2.5%) and rinsed by phosphate buffered saline (PBS) powder (pH 7.4). We applied Alexa Fluor^®^ 488 conjugate of concanavalin A (Thermo Fisher Scientific, China), which could be excited with a 488 nm laser line and detected with a BP (band-pass) 500–530 nm filter, to label α-mannopyranosyl and α-glucopyranosyl residues in extracellular polysaccharides constituting the basic structure of root biofilm.

## Results

### Distinctive Molecular Ecological Network Structure and Connectivity Within Four Compartments Across the Soil-Mangrove Root Interface

**FIGURE 1:**
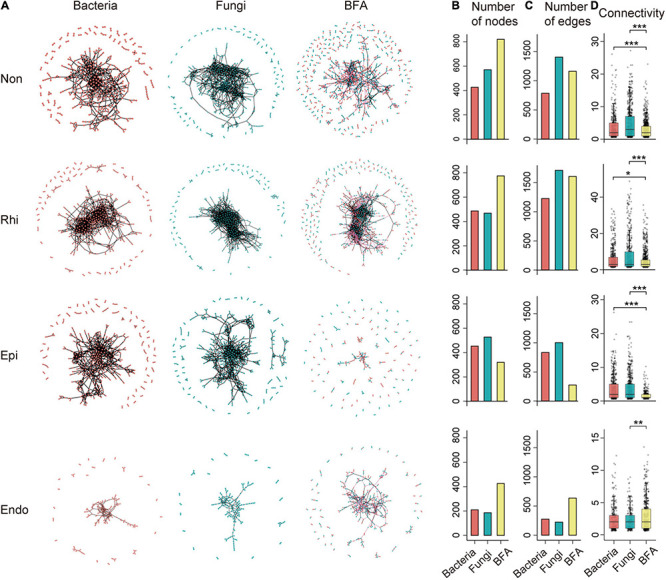
The profile of intra-domain and inter-domain networks across the non-rhizosphere (Non), rhizosphere (Rhi), episphere (Epi), and endosphere (Endo) compartments. **(A)** A representation of bacterial, fungal and bacterial-fungal association (BFA) networks across four compartments in the soil-mangrove root system. Each node represents an operational taxonomic unit (OTU), and each edge represents a correlation between two OTUs. Red and blue nodes represent bacteria and fungi, respectively, whereas black and pink edges represent intra-domain and inter-domain interactions, respectively. **(B,C)** Show the total number of nodes and edges for each network. **(D)** The distribution of connectivity harbored by each node in bacterial, fungal and BFA networks. Connectivity is measured by degrees, which refers to the number of edges one node links with. Significance is tested with a two-sided Fisher’s exact test: *P < 0.05; **P < 0.01; ***P < 0.001.

To explore putative microbe-microbe interactions across the soil-mangrove root interface, we analyzed microbial intra-domain and inter-domain co-occurrences based on sequencing datasets of bacterial and fungal communities (Figure 1A and Supplementary Table 3), which were represented as single bacterial and fungal network, and bacterial-fungal association (BFA) networks. Across four compartments in the soil-root system, we obtained a total of 12 microbial co-occurrence networks and their *R*^2^ of power-law ranged from 0.860 to 0.929 (Supplementary Table 4), suggesting that those network connectivity distributions were fitted well with the scale-free property (Deng et al., 2012).

Multiple network topological metrics consistently revealed that microbial co-occurrence patterns differed profoundly across four compartments (Figure 1). The assemblages of three peripheral compartments (non-rhizosphere, rhizosphere and episphere) formed larger and more complex networks with more nodes and edges than that of the endosphere, especially for BFA (Figures 1B,C). More interestingly, network complexity also differed between microbial intra-domain and inter-domain co-occurrence networks. The BFA networks exhibited a lower average connectivity than their corresponding bacterial and fungal networks in three peripheral compartments (Figure 1D). However, in the endosphere, a significantly (*P* < 0.05, two-tailed Fisher’s exact test) higher average connectivity was detected in the BFA network (2.986) than in the fungal (2.419) network (Figure 1D and Supplementary Table 4), and more nodes and edges were also observed in the BFA network (426 and 636) than in the single bacterial or fungal networks (210 and 276 for bacteria; 186 and 225 for fungi) (Figures 1B,C). The results revealed that the endosphere was the only compartment in which the BFA network harbored a denser co-occurrence pattern than the single bacterial or fungal networks, creating a distinctive niche inside root with the enhanced microbial inter-domain associations.

We next identified inter-domain microbial assemblages that potentially interacted or shared niches across four compartments in the soil-root system. Four representative BFA networks contained modules with modularity >0.5 (Supplementary Table 4), and we concentrated on modules with more than 15 nodes. Similar to the overall BFA network structure (Figure 1A), the number of modules became smaller in transition from the non-rhizosphere, rhizosphere to episphere, but became larger again in the endosphere (Supplementary Figure 1). Notably, we found that module composition did not show clear differences across four compartments, in which the proportions of bacteria and fungi were nearly identical (Supplementary Figure 1). The results indicated that the bacteria and fungi contributed similar proportion to the microbial inter-domain associations in the soil-mangrove root continuum.

### The Profile of Keystone Taxa Varied Across the Soil-Mangrove Root Interface

**FIGURE 2:**
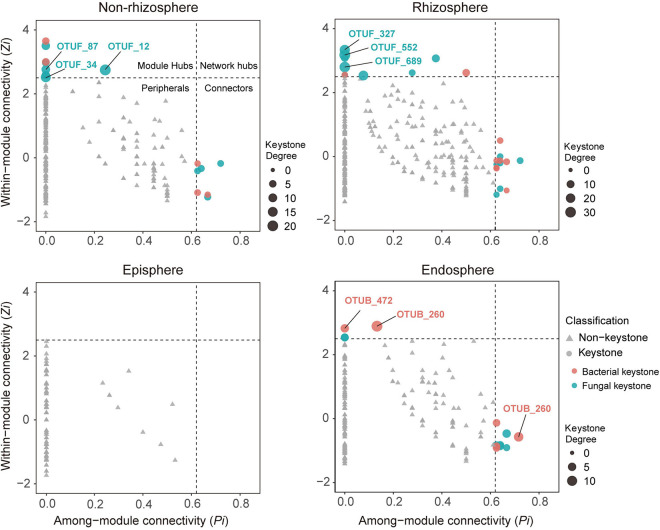
Topological features of keystone taxa in bacterial-fungal association (BFA) networks across four compartments in the soil-mangrove root system. Each bacterial operational taxonomic unit (OTU) is represented as OTUB, and each fungal OTU is represented as OTUF. Module hubs have within-module connectivity (*Zi*) > 2.5, whereas connectors have among-module connectivity (*Pi*) > 0.62. Keystone taxa and their domains are classified by shape and color, respectively. The size of each node indicates its degrees; keystone taxa with top three highest degrees in each compartment are labeled.

On the basis of within-module connectivity (*Zi*) and among-module connectivity (*Pi*) values, we classified all network nodes into four parts: module hubs, network hubs, peripherals and connectors (Shi et al., 2016). Due to the roles in network topology, the taxa detected as module hubs, network hubs and connectors were proposed to be keystone taxa in the complex microbial communities. The results showed that most nodes from each network were peripherals, and no network hubs were identified (Figure 2). Intriguingly, in the episphere, more module hubs and connectors were detected in the single bacterial (14) and fungal (13) networks than in the BFA (0) networks (Supplementary Figure 2); however, in the endosphere compartment, the corresponding BFA network harbored more keystone taxa (Supplementary Figure 2), which is consistent with its more complicated networks (Figure 1A).

To investigate the role of keystone taxa in microbial inter-domain associations, we focused on BFA networks and their associated keystone taxa across four compartments in the soil-root system. We found that 15, 23, and 13 keystone taxa were observed in the BFA networks of the non-rhizosphere, rhizosphere, and endosphere, respectively, but no keystone taxa were detected in the episphere (Figure 2). Consistently, most of these putative keystone taxa had low relative abundances (Supplementary Figure 2 and Supplementary Table 3), suggesting that low-abundant taxa may significantly contribute to mangrove root functions. Further analysis of keystone taxa indicated that fungi accounted for a larger proportion (60.5%) in module hubs and connectors of BFA networks than bacteria (39.5%). Nonetheless, they displayed distinct importance across those four compartments. In the non-rhizosphere and rhizosphere, keystone taxa with top three highest degrees (the number of links for a particular node) were all monopolized by fungi (Supplementary Figure 3). However, in the endosphere, keystone taxa with top three highest degrees belonged to bacteria, and they were affiliated with *Vibrio* (OTUB_1491), *Anaerolineae* (OTUB_472) and *Desulfarculaceae* (OTUB_260) (Figure 2 and Supplementary Table 3).

### Genetic Divergence of Quorum Sensing and Cobamide Biosynthesis Across the Soil-Mangrove Root Interface

In order to understand possible mechanisms of microbial interactions across soil-root interface, we investigated the functional profile of microbial communities by shotgun metagenome sequencing. We focused on functional genes and pathways involved in quorum sensing and cobamide biosynthesis as they have been considered as typical strategies of microbial interactions possessed by various communities (Milton et al., 1997; Olga et al., 2020).

**FIGURE 3:**
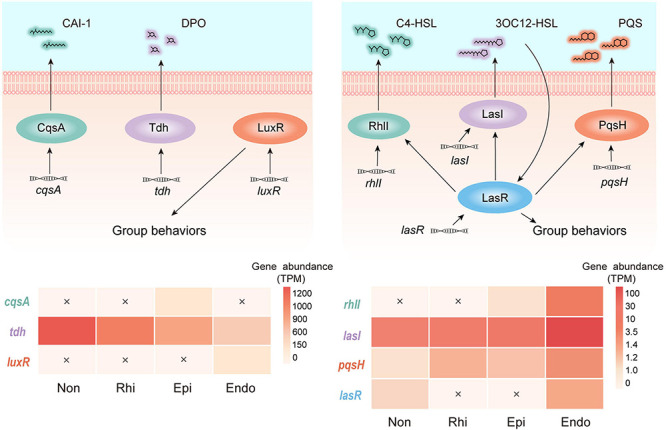
Profiles of two quorum sensing modules across the non-rhizosphere (Non), rhizosphere (Rhi), episphere (Epi) and endosphere (Endo) compartments. In module 1, *cqsA* and *tdh* encode the autoinducers (S)-3-hydroxytridecan-4-one (CAI-1) and 3,5-dimethyl-pyrazin-2-ol (DPO), respectively, and *luxR* mediates group behaviors. In module 2, *lasI*, *rhlI*, and *pqsH* encode the autoinducers N-(3-oxododecanoyl)-L-homoserine lactone (3OC12-HSL), N-butyryl-L-homoserine lactone (C4-HSL), and 2-heptyl-3-hydroxy-4-quinolone (PQS), respectively. *lasR* encodes the receptor of 3OC12-HSL which mediates group behaviors. The relative abundance (TPM, transcripts per-million) of genes belonging to each module across four compartments in the soil-mangrove root system is represented by heatmaps, and each undetected gene is marked with a cross.

Quorum sensing served as a cell-cell communication device, which was exploited by many microflora (Milton et al., 1997). Here, we detected quorum sensing circuits with two modules represented by Gram-negative bacteria, and found that genes involved in quorum sensing were unevenly distributed across four continuous compartments (Figure 3). In module 1, the genes individually related to the production of autoinducer (S)-3-hydroxytridecan-4-one (CAI-1) and the mediation of group behaviors, *cqsA*, and *luxR*, were highly abundant in the episphere and endosphere, respectively. As the most abundant gene in module 1, *tdh* relates to the production of autoinducer 3,5-dimethyl-pyrazin-2-ol (DPO) and its abundance continuously decreased from the non-rhizosphere to the endosphere. In module 2, *lasI* and *pqsH*, related to production of autoinducer N-(3-oxododecanoyl)-L-homoserine lactone (3OC12-HSL) and 2-heptyl-3-hydroxy-4-quinolone (PQS), were detected across four compartments, but showed a relatively higher abundance in the endosphere. More strikingly, the endosphere was the only compartment that simultaneously contained *rhlI* and *lasR*, which were involved into the syntheses of autoinducers N-butyryl-L-homoserine lactone (C4-HSL) and the mediation of group behaviors, respectively. The divergent pattern of genes involved in quorum sensing indicated that three peripheral compartments held a greater potential in conducting module 1, whereas module 2 was more likely to be conducted in the endosphere (Figure 3).

**FIGURE 4:**
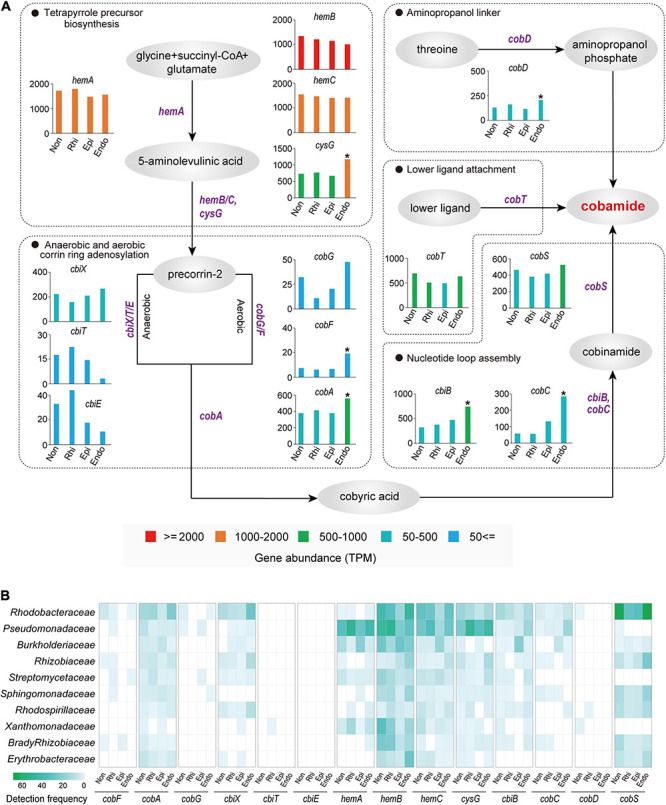
Cobamide biosynthesis pathways and taxonomy profiles of cobamide biosynthesis microbiomes across the non-rhizosphere (Non), rhizosphere (Rhi), episphere (Epi), and endosphere (Endo) compartments. **(A)** Each substance is indicated by a gray oval, whereas each functional gene is labeled in purple. The significantly higher TPM (transcripts per-million) value of gene in the endosphere is indicated by an asterisk (*P* < 0.05; two-tailed fisher exact test), compared to the three peripheral compartments. **(B)** Bacterial families with the top 10 highest detection frequency in cobamide biosynthesis genes are exhibited by a heatmap.

Cobamide, as one of the sharing valuable nutrients, was widely used by microbes to interact with each other (Olga et al., 2020). Our metagenome sequencing analysis revealed that among 15 cobamide biosynthesis genes, six genes (*cysG*, *cobF*, *cbiB*, *cobC*, *cobA*, and *cobD*) were significantly (*P* < 0.05, two-tailed Fisher’s exact test) higher in the endosphere than in other three compartments, and they were involved in each of key steps for cobamide biosynthesis, including the tetrapyrrole precursor biosynthesis, corrin ring adenosylation, nucleotide loop assembly and aminopropanol phosphate production (Figure 4A). Intriguingly, a compartment specificity was detected for anaerobic or aerobic corrin ring adenosylation. Compared to three peripheral compartments, the endosphere harbored a higher abundance of genes associated with aerobic adenosylation (*cobG*, *cobF*, and *cobA*) and a lower abundance of genes associated with anaerobic adenosylation (*cbiT* and *cbiE*) (Figure 4A). Furthermore, the taxonomic composition of all cobamide-related genes across four compartments (Figure 4B) revealed a high detection frequency of *Rhodobacteraceae* and *Pseudomonadaceae*, highlighting their key roles in mediating microbial interactions by producing cobamides. It is important to note that these two families were characterized by a nearly equal detection frequency across four compartments in the soil-root system (Figure 4B), with a low abundance (2.98–6.81%) in the whole bacterial community (Supplementary Figure 4). Therefore, these results indicated the intensified microbial associations in the endosphere were linked to aerobic cobamide biosynthesis performed by specialists (*Rhodobacteraceae* and *Pseudomonadaceae*). Apart from these two families, other cobamide-producing members (e.g., *Rhizobiaceae*, *Sphingomonadaceae*, and *Rhodospirillaceae*; Figure 4B) also played non-negligible roles in mediating microbial associations since we detected them as the keystone taxa in microbial networks (Supplementary Table 3).

### Metagenomic, Microscopic, and Root Exudate Analyses Provide Evidences for Biofilm Formation in the Endosphere

**FIGURE 5:**
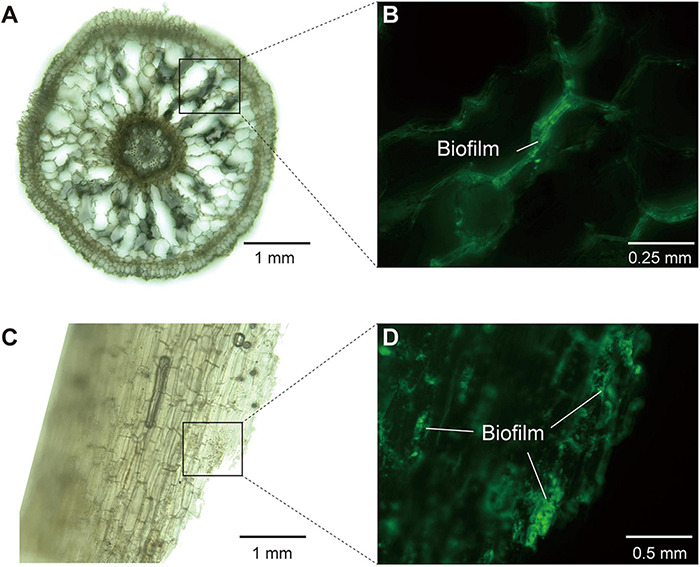
The structure of mangrove roots and the detection of biofilm in root interior under hybrid microscope. **(A,B)** The images of longitudinal section of mangrove roots (×10 and ×20 magnification, respectively). **(C,D)** The images of transverse section of mangrove roots (×10 and ×40 magnification, respectively). **(A,C)** Were in brightfield, whereas **(B,D)** were in fluorescence field (488 nm, Mercury Free LED) with extracellular polysaccharides in biofilm being stained and glowing green light.

To further consolidate the existence of biofilm in the endosphere compartment of mangrove roots, we provided multiple lines of evidence based on metagenomic, microscopic and root exudate analyses. First, our metagenomic data determined that the endosphere contained high abundance of KOs related to the biosynthesis of extracellular polymeric substances (EPS) (Supplementary Figure 5), which were well known as structural and functional elements crucial to the formation of biofilms (Karygianni et al., 2020). Second, microscopic analysis confirmed the occurrence of biofilm inside mangrove roots, since we detected α-mannopyranosyl and α-glucopyranosyl residues that constituted the basic structure of EPS in root biofilm (Figure 5). Third, through root exudate analysis with LC/MS, we detected two EPS compounds yielding from the mangrove root interior, and they were trehalose with median retention time at 147.22 s (negative mode) and 149.87 (positive mode), and glucans with median retention time at 48.13 s (negative mode) and 345.06 s (positive mode) (Supplementary Table 5). In addition, we also detected adenine with median retention time at 107.68 s (negative mode) and 149.88 s (positive mode) as the component of root exudates, and this substance was reported to participate in the biosynthesis of lower ligand structure of cobamide (Shelton et al., 2019). Collectively, these results from multiple assays demonstrated the occurrence of biofilm and cobamide inside root, which built foundations for the intensified microbial associations in the endosphere of mangrove roots.

## Discussion

Understanding the network structure of microbial communities is essential to decipher their putative interactions and ecological roles (Zhou et al., 2010; Yuan et al., 2021). Through network topology and analysis of genes related to quorum sensing and cobamide biosynthesis, we found niche differentiation of microbial associations and keystone taxa across the soil-mangrove root interface. Compared to three peripheral compartments, the endosphere exhibited an intensified association between bacterial and fungal communities, which was largely attributable to the specific keystone taxa and distinctive interaction strategies.

Given the essential topological role of keystone taxa in microbial networks (Ma et al., 2018; Fan et al., 2020), previous studies have determined the importance of keystone taxa in regulating microbial interactions. In this study, keystone taxa showed a clear divergence across the soil-mangrove root interface. In the endosphere, the bacterial keystone taxa with top three highest degrees were likely to have a role in enhancing inter-domain microbial associations, validating their important status in bacterial-fungal co-occurrence networks. OTUB_260 was affiliated with the *Vibrio* genus, and its members were capable of inhibiting fungal phytopathogens by releasing antimicrobial compounds, thereby conducting negative bacterial-fungal associations (Rameshkumar and Nair, 2009). OTUB_472, was affiliated to *Anaerolineae*, and the members of this class were known to accelerate organic matter degradation when they were stimulated by hyphae exudates of arbuscular mycorrhizal fungi (Cao et al., 2018; Toju and Sato, 2018). Additionally, OTUB_1491 was a member in *Desulfarculaceae* family, and these typical sulfate-reducing bacteria were reported to act as nutrient providers for fungi and trigger fungal colonization (Drake et al., 2017; He et al., 2019). Apart from the natural feature of keystones, biofilm also played an important role in regulating microbial interactions. Early evidence showed that biofilm could form in mangrove roots (Mallick et al., 2018; Glasenapp et al., 2019). Combined with the fact that endophytes were capable of forming mixed-community biofilms inside roots (van Overbeek and Saikkonen, 2016), we supposed a high potential of the mixed-community biofilm formation in the endosphere of the mangrove root, thereby providing a niche for intensive microbial inter-domain associations (Cai et al., 2019). In support of this view, we found that the endosphere contained a high abundance of KOs related to the biosynthesis of EPS, and was capable of producing two EPS compounds (trehalose and glucans) and forming biofilms. More importantly, we identified one keystone taxa (OTUB_260) belong to *Vibrio* sp., which was reported to promote microbial biofilm formation when it communicated with fungi and utilized volatile organic compounds produced by fungi (Cosetta et al., 2020). Correspondingly, these results indicated that the intensified inter-domain microbial associations in the endosphere were related to the specific keystone composition and biofilm formation.

Quorum sensing controls biofilm formation and has been established as a widespread strategy of cell-cell communications in microbial communities (Kim et al., 2017; Padder et al., 2018). In the module 1 we detected in this study (Figure 3), the simultaneous occurrence of *tdh* and *cqsA* may be attributable to the weak microbial inter-domain associations in the episphere. This is because the autoinducers encoded by these two genes were considered to inhibit biofilm formation (Zhu and Mekalanos, 2003; Papenfort et al., 2017) and hence have the potential to hamper microbial cell-cell communications. On the contrary, the module 2 of quorum sensing tended to play a major role in the endopshere, where its involved genes (*rhlI* and *lasI*) were highly abundant. The autoinducers C4-HSL and 3OC12-HSL that were regulated by *rhlI* and *lasI*, respectively, were reported to facilitate the establishment of biofilms in the endosphere (Ozer et al., 2005), potentially leading to the divergent microbial associations across the soil-mangrove root interface. Besides, the level of quorum sensing autoinducers was related to the variations in environmental factors. Through our mantel test, we found that salinity, temperature and oxidation reduction potential significantly affected (*P* < 0.01) the composition of bacteria and fungi communities in mangrove soils (Supplementary Figure 6). Among these influencing factors, salinity was previously known to play an essential role in affecting the root-associated microbial communities of mangrove (Yin et al., 2018) and the stability of quorum sensing molecules (Huang et al., 2018). Hence, we indicated that environmental factors, especially salinity, could influence quorum sensing autoinducers via shifting mangrove root-associated microbial communities.

Similar to quorum sensing, cobamide has been also widely used as model nutrients for studying microbial interactions (Olga et al., 2020). It was established that cobamide mediated microbial interactions through cobamide-dependent metabolism in a metabolic network elicited by cobamide-dependent reactions (Olga et al., 2020). As the most well-known cobamide, vitamin B12 produced by bacteria was reported to have stimulatory effect on mycorrhizal growth in the rhizosphere soil (Singh et al., 1991), highlighting mutualistic interactions between bacteria and their eukaryotic partners (van Overbeek and Saikkonen, 2016). In this study, we detected adenines within root exudates and the functional genes involving into each step of cobamide biosynthesis, proposing the functional genes involving into each step of cobamide biosynthesis, and proposed *Rhodobacteraceae* and *Pseudomonadaceae* as the key taxa for cobamide synthesis across soil-mangrove root interface. Their crucial roles in synthesizing cobamide for mediating microbial interactions have been documented in artificial seawater (Wienhausen, 2018). Through metagenome sequencing analysis across four compartments in the soil-root system, we surprisingly found that the endosphere contained a high abundance of cobamide biosynthesis related genes, and there are two reasons for such enrichment inside plant roots. First, abiotic factors (e.g., oxygen) might contribute to the discrepancy of cobamide biosynthesis between outside and inside roots. Due to the tidal process in mangrove ecosystems (Wang et al., 2014), a relatively anoxic circumstance probably existed in peripheral compartments (Wang et al., 2014). This could explain why we detected a high abundance of *cbiT* and *cbiE* encoding for anaerobic corrin ring adenosylation in three peripheral compartments. On the contrary, mangroves were thought to contain large lacunae in the cortex to efficiently transfer internal oxygen, thereby preventing oxygen loss and maintaining aerobic inside the root environment (Pi et al., 2009). As a function of such aerobic condition, we observed that the endosphere harbored a high abundance of *cobG*, *cobF* and *cobA* encoding for aerobic corrin ring adenosylation. Second, cobamide utilization can be mediated by quorum sensing molecules. Previous studies have indicated that certain quorum sensing autoinducers, such as 3OC12-HSL from the module 2, would stimulate microbial communities to accumulate cobamides and subsequently promote microbial growth (Johnson et al., 2016; Das and Ghangrekar, 2019). In support of this hypothesis, we detected the concurrently enriched genes encoding both the module 2 of quorum sensing and cobamide biosynthesis in the endosphere, which coincided well with intensified associations between bacterial and fungal communities.

**FIGURE 6:**
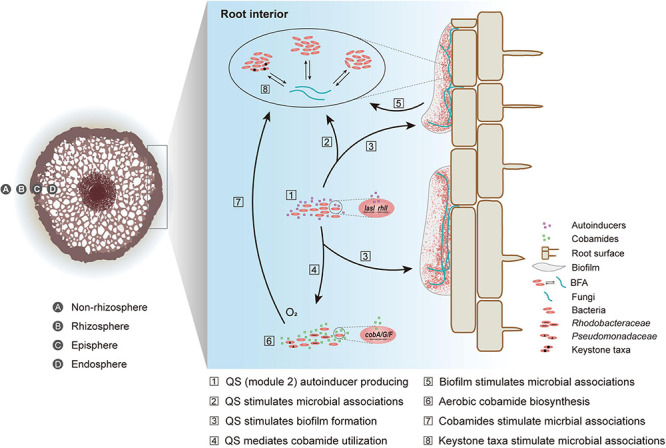
A conceptual model of the intensified bacterial-fungal association (BFA) in root interior. Quorum sensing is abbreviated to QS.

Based on our findings and relevant studies (Bertrand et al., 2015; Banerjee et al., 2018; Huang et al., 2018; Shelton et al., 2019; Olga et al., 2020), we built a conception model to illustrate how microbial inter-domain interactions were enhanced in the endosphere of mangrove roots (Figure 6). First, through network topological analysis and metagenomic function survey, we found that BFA was divergent across four mangrove root compartments. The intensified BFA in the endosphere could be attributed to the three bacterial keystones (*Vibrio*, *Anaerolineae*, and *Desulfarculaceae*). This was because these bacterial taxa have been thought to closely react with fungal members (Drake et al., 2017; Cao et al., 2018; Toju and Sato, 2018; He et al., 2019; Cosetta et al., 2020). Second, compared to three peripheral compartments, the endosphere contained a higher abundance of genes related to module 2 of quorum sensing and aerobic cobamide biosynthesis, which was consolidated by root exudate analysis. Such enrichment could contribute to the intensified BFA in the endosphere (Bertrand et al., 2015; Banerjee et al., 2018; Huang et al., 2018; Shelton et al., 2019; Olga et al., 2020). Third, quorum sensing could indirectly regulate microbial interactions via mediating cobamide utilization (Johnson et al., 2016; Das and Ghangrekar, 2019) and stimulating biofilm formation (Miller and Bassler, 2001; Kim et al., 2017). Last, the high abundance of EPS-related genes, the production of two EPS compounds within root exudates and the detection of extracellular polysaccharides in the endosphere collectively suggested the existence of biofilm inside mangrove root, which provided a niche for endophytes to develop intensive microbial inter-domain associations. While this study is the first to molecularly characterize the roles of quorum sensing and cobamide biosynthesis across the soil-mangrove root interface, the underlying mechanism at biochemical and phenotypical scales needs further investigation.

## Conclusion

Through topological and metagenomic analyses of microbial communities across four compartments in the soil-root system, we profiled the varied bacterial-fungal associations across the soil-mangrove root interface. Particularly, we found that the bacterial keystone taxa, the mediation of quorum sensing (module 2), biofilm formation as well as the aerobic cobamide biosynthesis by *Rhodobacteraceae* and *Pseudomonadaceae* might jointly contribute to the intensified bacterial-fungal associations in the endosphere. On the whole, our study provides the genetic elucidation of quorum sensing and cobamide biosynthesis in divergent bacterial-fungal associations across the soil-root interface.

## Data Availability Statement

The datasets presented in this study including nucleotide sequences and metagenome sequencing data can be found in online SRA repositories with accession numbers as PRJNA685020, PRJNA685297 and PRJNA613873, respectively.

## Author Contributions

ZZ: investigation, data curation, formal analysis, and writing-original draft. RH, YN, WH, and QZ: methodology, writing-review, and editing. WZ and ZL: investigation, writing-review, and editing. QY: methodology and funding acquisition. ZH: methodology, supervision, and funding acquisition. CW: conceptualization, supervision, funding acquisition, writing-review, and editing. All authors contributed to the article and approved the submitted version.

## Conflict of Interest

The authors declare that the research was conducted in the absence of any commercial or financial relationships that could be construed as a potential conflict of interest.

## Publisher’s Note

All claims expressed in this article are solely those of the authors and do not necessarily represent those of their affiliated organizations, or those of the publisher, the editors and the reviewers. Any product that may be evaluated in this article, or claim that may be made by its manufacturer, is not guaranteed or endorsed by the publisher.

## Footnotes

1
http://ieg2.ou.edu/MENA/

## Supplementary Material

Supplementary Figure 1 Highly connected modules within bacterial-fungal association (BFA) networks across the non-rhizosphere (Non), rhizosphere (Rhi), episphere (Epi), and endosphere (Endo) compartments.

Supplementary Figure 2 Topological features of keystone taxa in **(A)** bacterial, **(B)** fungal and **(C)** bacterial-fungal association (BFA) networks across the non-rhizosphere (Non), rhizosphere (Rhi), episphere (Epi), and endosphere (Endo) compartments.

Supplementary Figure 3 Degrees of keystone taxa from bacterial and fungal phyla in bacterial-fungal association (BFA) networks.

Supplementary Figure 4 Bacterial community compositions across the non-rhizosphere (Non), rhizosphere (Rhi), episphere (Epi), and endosphere (Endo) compartments.

Supplementary Figure 5 The relative abundance (TPM, transcripts per-million) of KOs (KEGG Orthology) that related to the biosynthesis of extracellular polymeric substances (EPS) across the non-rhizosphere (Non), rhizosphere (Rhi), episphere (Epi), and endosphere (Endo) compartments.

Supplementary Figure 6 Correlations of the microbial communities (Bray-Curtis distance) with environmental factors (Euclidean distance) in the non-rhizosphere soil. Edge width corresponded to the Mantel’s *r*-value, and the edge color denoted the statistical significance. Pairwise correlations of the variables were shown with a color gradient denoting Pearson’s correlation coefficient.

Supplementary Table 1 Environmental factors in the non-rhizosphere soil of mangrove.

Supplementary Table 2 KO (KEGG Orthology) numbers of quorum sensing and cobamide biosynthesis related genes.

Supplementary Table 3 Taxonomy information and relative abundance of keystone taxa in bacterial-fungal association (BFA), bacterial and fungal networks across the non-rhizosphere (Non), rhizosphere (Rhi), episphere (Epi), and endosphere (Endo) compartments.

Supplementary Table 4 Key network topological characteristics for bacterial, fungal and bacterial-fungal association (BFA) networks across the non-rhizosphere (Non), rhizosphere (Rhi), episphere (Epi), and endosphere (Endo) compartments.

Supplementary Table 5 Root exudates that related to biofilm formation and cobamide biosynthesis.
