# Supplementary material for: Genetic Elucidation of Quorum Sensing and Cobamide Biosynthesis in Divergent Bacterial-Fungal Associations Across the Soil-Mangrove Root Interface
Source: Front Microbiol. 2021 Oct 5;12:698385. doi: 10.3389/fmicb.2021.698385 (PMC8524053; doi:10.3389/fmicb.2021.698385)
Supplement: Supplementary Figure 1 — Highly connected modules within bacterial-fungal association (BFA) networks across the non-rhizosphere (Non), rhizosphere (Rhi), episphere (Epi), and endosphere (Endo) compartments. [file Data_Sheet_1.pdf]

SUPPLEMENTARY MATERIAL for:

Genetic elucidation of quorum sensing and cobamide biosynthesis in divergent bacterial-fungal associations across the soil-mangrove root interface

Zhengyuan Zhou[1], Ruiwen Hu[1], Yanmei Ni[2], Wei Zhuang[1], Zhiwen Luo[1], Weiming Huang[1], Qingyun Yan[1], Zhili He[1], Qiuping Zhong[1], Cheng Wang[1*]

[1]Environmental Microbiomics Research Center, School of Environmental Science and Engineering, Southern Marine Science and Engineering Guangdong Laboratory (Zhuhai), Sun Yat-sen University, Guangzhou 510006, China

[2]Guangdong Agribusiness Tropical Agriculture Institute, Guangzhou 510006, China

***Correspondence author:**

Cheng Wang

Tel：+86 020 84113485

E-mail: wangcheng5@mail.sysu.edu.cn

SUPPLEMENTARY MATERIAL

FIGURE S1. Highly connected modules within bacterial-fungal association (BFA) networks across the non-rhizosphere (Non), rhizosphere (Rhi), episphere (Epi) and endosphere (Endo) compartments.

FIGURE S2. Topological features of keystone taxa in **(A)** bacterial, **(B)** fungal and **(C)** bacterial-fungal association (BFA) networks across the non-rhizosphere (Non), rhizosphere (Rhi), episphere (Epi) and endosphere (Endo) compartments.

FIGURE S3. Degrees of keystone taxa from bacterial and fungal phyla in bacterial-fungal association (BFA) networks.

FIGURE S4. Bacterial community compositions across the non-rhizosphere (Non), rhizosphere (Rhi), episphere (Epi) and endosphere (Endo) compartments.

FIGURE S5. The relative abundance (TPM, transcripts per-million) of KOs (KEGG Orthology) that related to the biosynthesis of extracellular polymeric substances (EPS) across the non-rhizosphere (Non), rhizosphere (Rhi), episphere (Epi) and endosphere (Endo) compartments.

FIGURE S6. Correlations of the microbial communities (Bray-Curtis distance) with environmental factors (Euclidean distance) in the non-rhizosphere soil. Edge width corresponded to the Mantel's r value, and the edge color denoted the statistical significance. Pairwise correlations of the variables were shown with a color gradient denoting Pearson's correlation coefficient.

TABLE S1. Environmental factors in the non-rhizosphere soil of mangrove.

TABLE S2. KO (KEGG Orthology) numbers of quorum sensing and cobamide biosynthesis related genes.

TABLE S3. Taxonomy information and relative abundance of keystone taxa in bacterial-fungal association (BFA), bacterial and fungal networks across the non-rhizosphere (Non), rhizosphere (Rhi), episphere (Epi) and endosphere (Endo) compartments.

TABLE S4. Key network topological characteristics for bacterial, fungal and bacterial-fungal association (BFA) networks across the non-rhizosphere (Non), rhizosphere (Rhi), episphere (Epi) and endosphere (Endo) compartments.

TABLE S5. Root exudates that related to biofilm formation and cobamide biosynthesis.

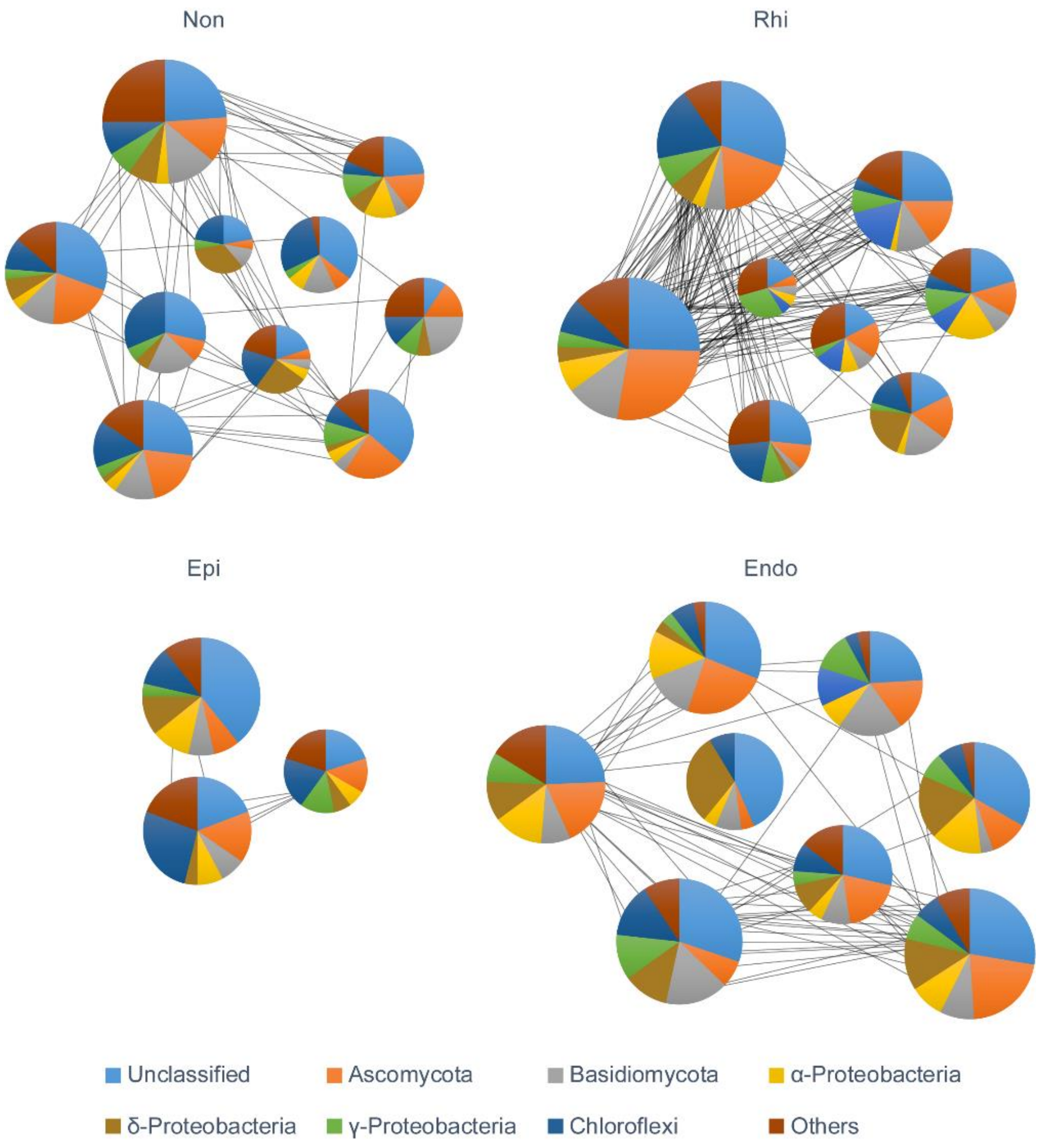

FIGURE S1. Highly connected modules within bacterial-fungal association (BFA) networks across the non-rhizosphere (Non), rhizosphere (Rhi), episphere (Epi) and endosphere (Endo) compartments. Edges represent interactions between different modules. Pie charts represent the modules containing more than 15 nodes, and colors indicate different major phyla.

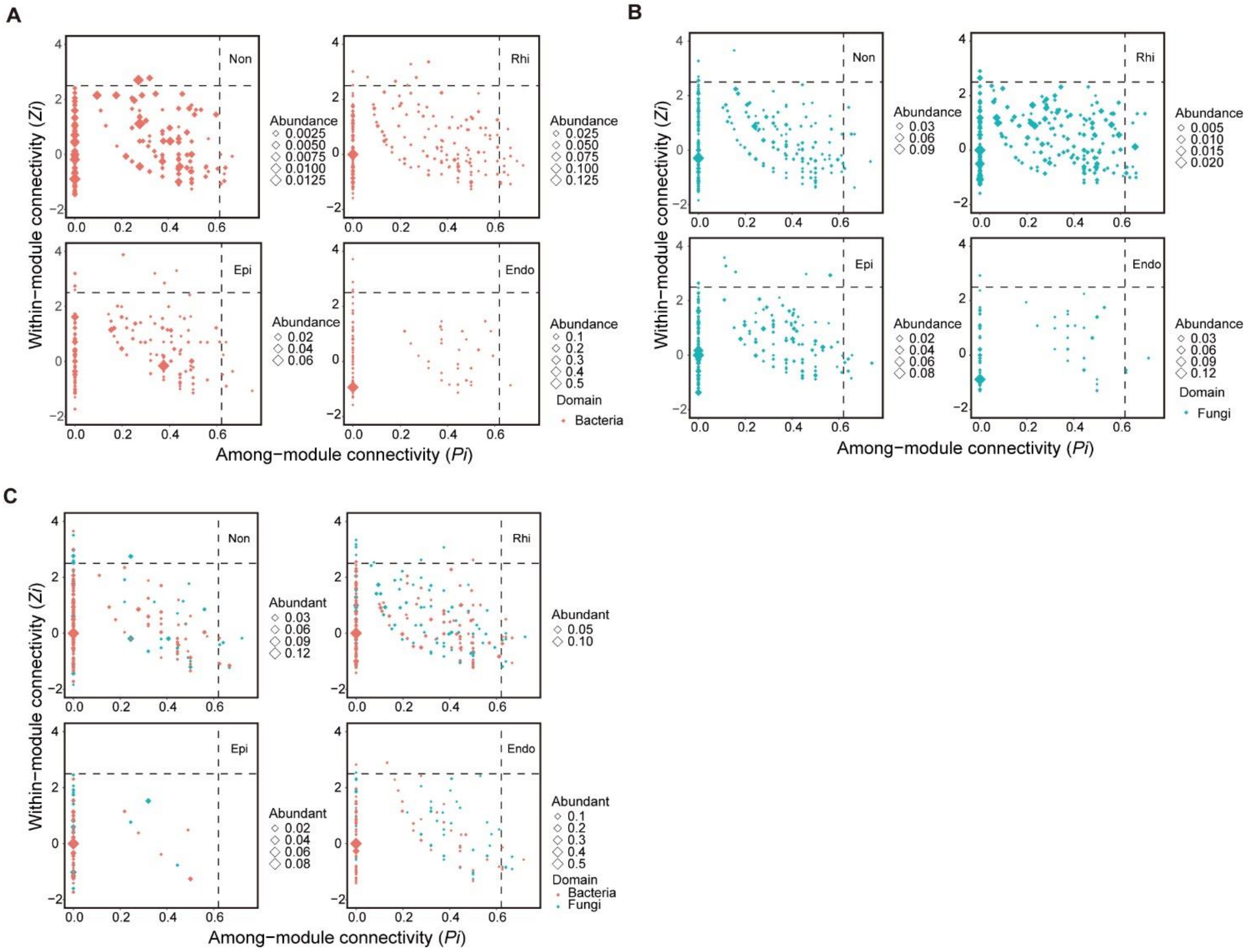

FIGURE S2. Topological features of keystone taxa in **(A)** bacterial, **(B)** fungal and **(C)** bacterial-fungal association (BFA) networks across the non-rhizosphere (Non), rhizosphere (Rhi), episphere (Epi) and endosphere (Endo) compartments. The size of each node is represented for each operational taxonomic unit (OTU)'s abundance.

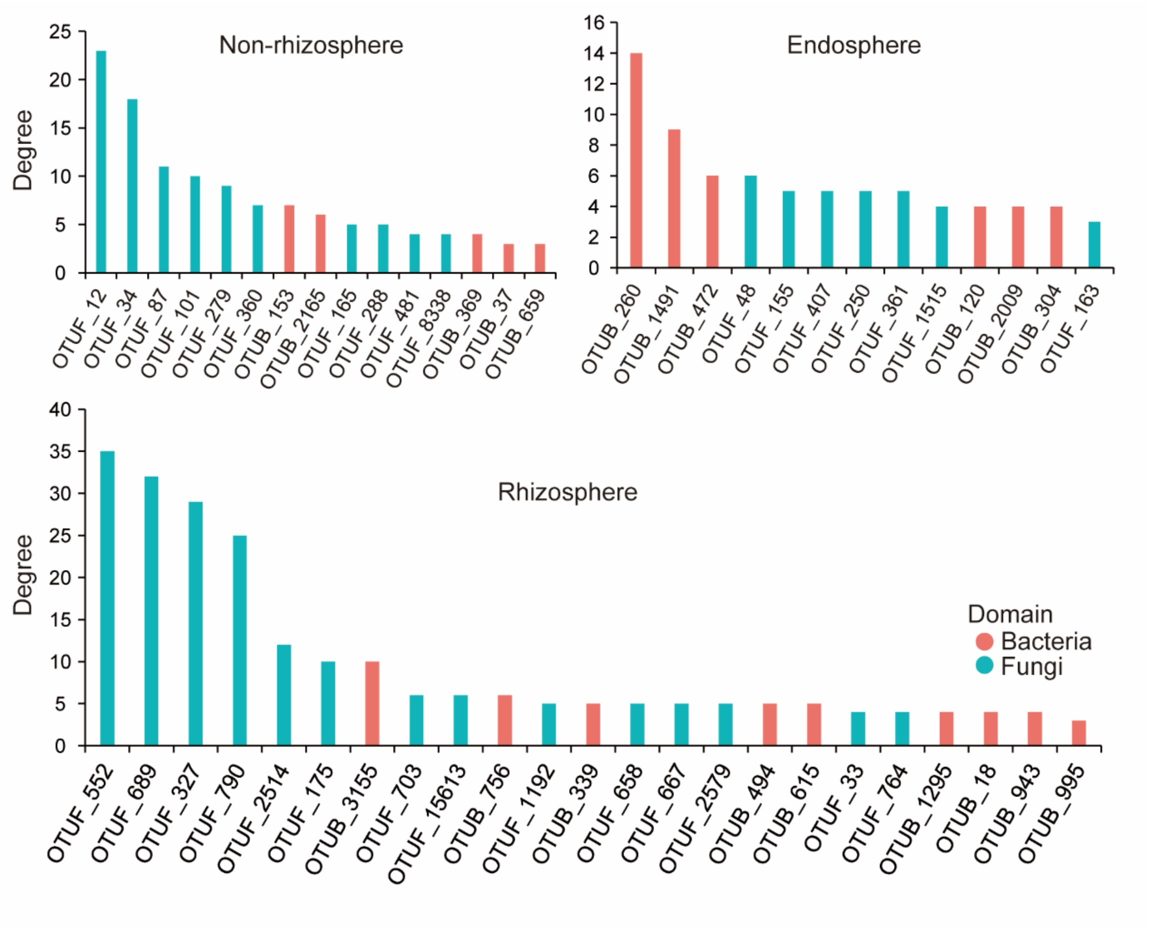

FIGURE S3. Degrees of keystone taxa from bacterial and fungal phyla in bacterial-fungal association (BFA) networks. Each bacterial operational taxonomic unit (OTU) is represented as OTUB, and each fungal OTU is represented as OTUF.

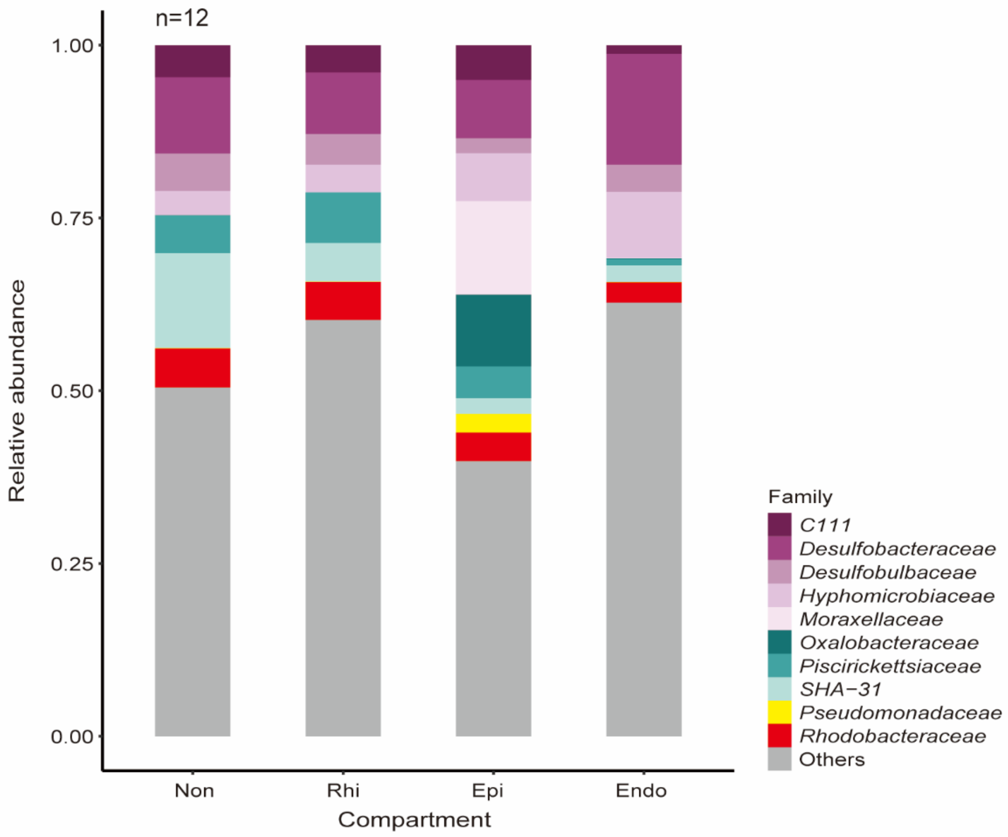

FIGURE S4. Bacterial community compositions across the non-rhizosphere (Non), rhizosphere (Rhi), episphere (Epi) and endosphere (Endo) compartments. Stacked bar chart shows the relative abundance of various families of bacterial communities, including *Rhodobacteraceae* and *Pseudomonadaceae*. The rest of more than 100 families are classified as others.

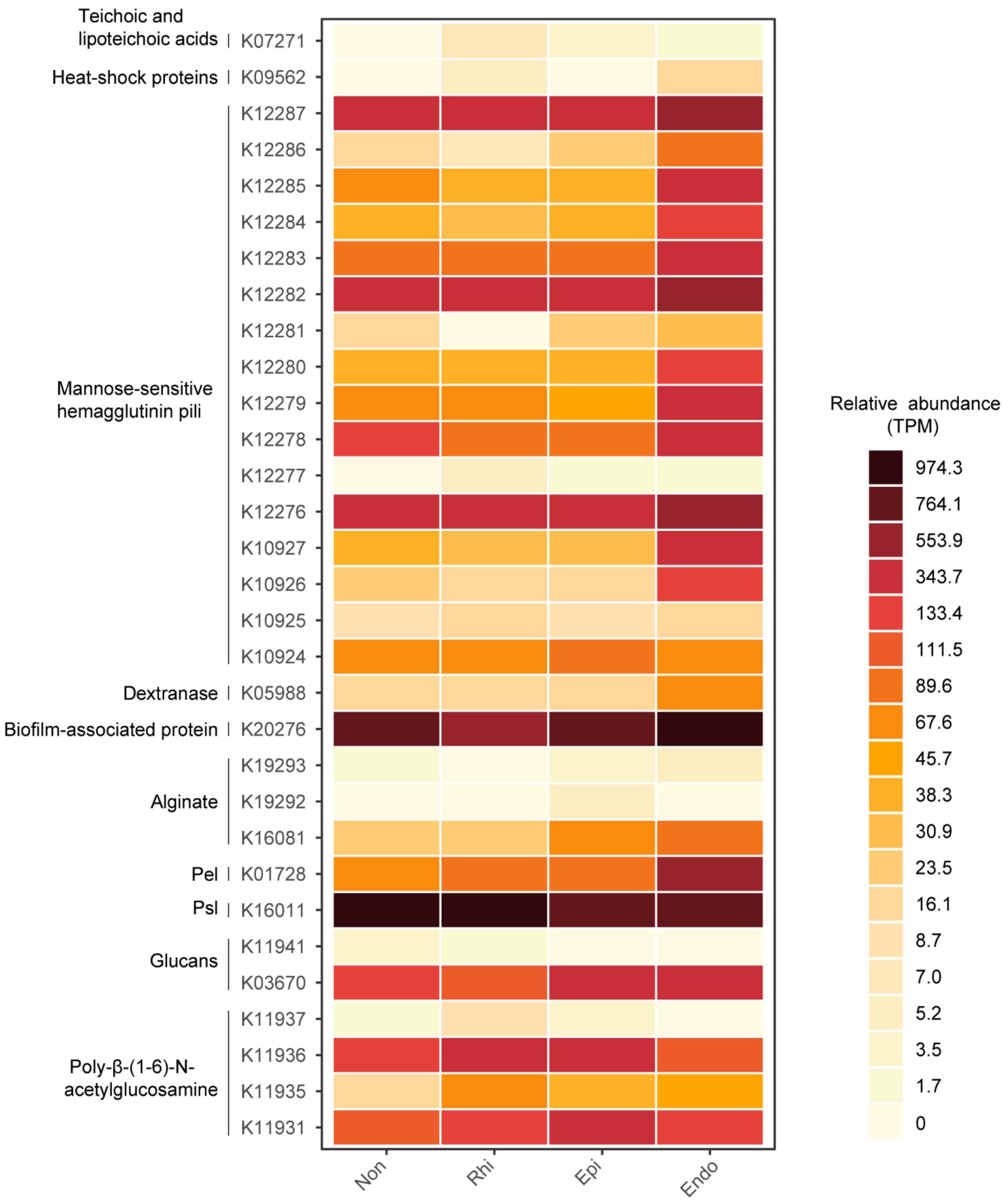

FIGURE S5. The relative abundance (TPM, transcripts per-million) of KOs (KEGG Orthology) that related to the biosynthesis of extracellular polymeric substances (EPS) across the non-rhizosphere (Non), rhizosphere (Rhi), episphere (Epi) and endosphere (Endo) compartments.

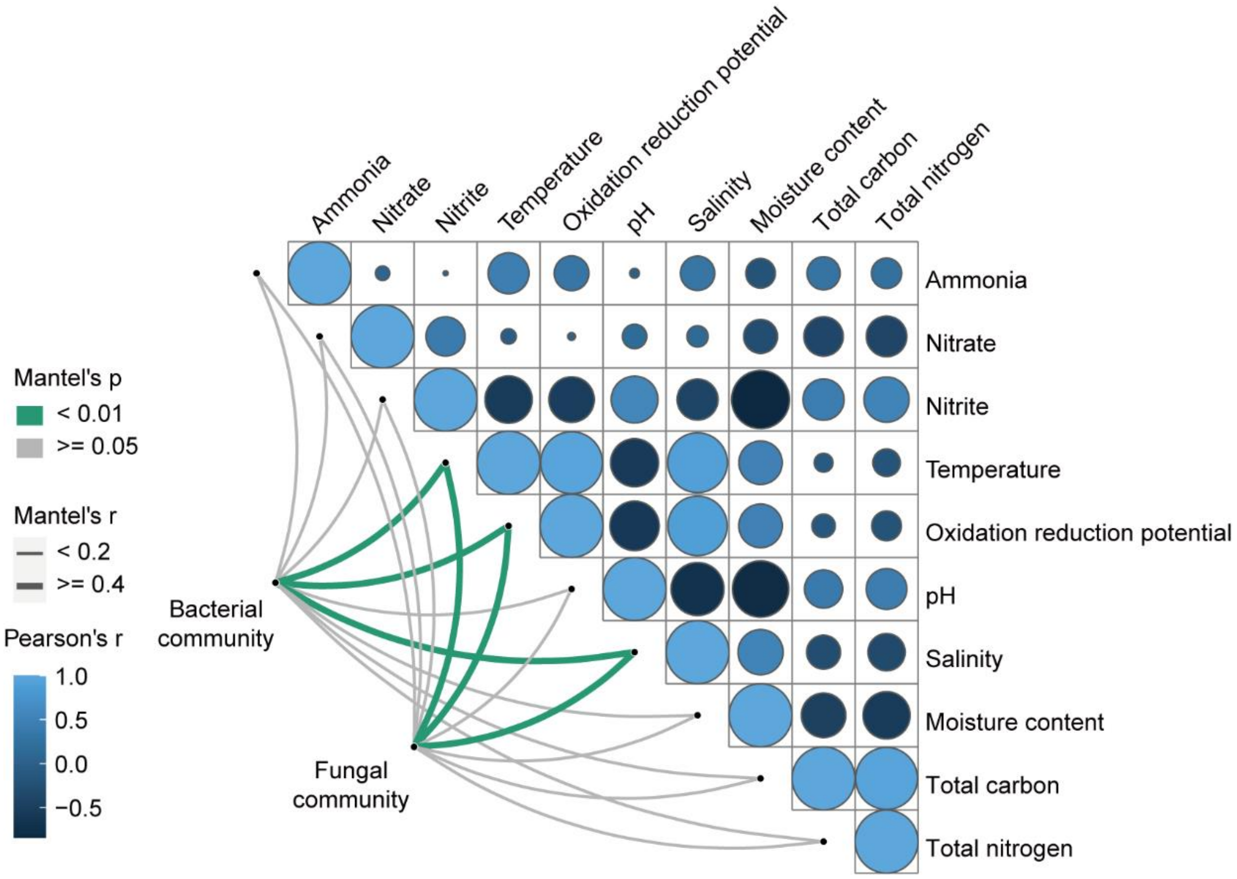

FIGURE S6. Correlations of the microbial communities (Bray-Curtis distance) with environmental factors (Euclidean distance) in the non-rhizosphere soil. Edge width corresponded to the Mantel's *r* value, and the edge color denoted the statistical significance. Pairwise correlations of the variables were shown with a color gradient denoting Pearson's correlation coefficient.

TABLE S1. Environmental factors in the non-rhizosphere soil of mangrove.

SampleID	Ammonia-N (mg/kg)	Nitrate-N (mg/kg)	Nitrite-N (mg/kg)	Temperature (℃)	Eh (mV)	pH	Salinity (‰)	Moisture contant (%)	Total carbon (mg/kg)	Total nitrogen (mg/kg)
S1	2.756491	0.292671	0.041408	28.3	-11	6.38	2.99	65.49865	8.26	0.14
S2	1.791578	0.251502	0.034804	28.7	-12	6.65	2.86	65.37087	9.39	0.14
S3	3.393989	0.301013	0.043416	28.7	-13	6.54	3.01	62.47736	8.65	0.18
S4	2.115601	0.205037	0.042647	28.3	-15	6.29	3.05	65.04642	15.02	0.46
S5	1.945985	0.236575	0.040138	28.4	-13	6.43	3.01	69.67213	15.87	0.55
S6	4.805075	0.203211	0.036097	28.8	-14	6.4	2.89	67.61811	27.41	0.69
S7	1.376528	0.254741	0.042621	26.9	-23	6.43	2.59	68.2384	7.52	0.16
S8	1.298231	0.243431	0.037105	26.8	-24	6.45	2.44	69.606	3.49	0.06
S9	2.609481	0.278305	0.043316	27.0	-25	6.79	2.69	61.07258	5.61	0.12
S10	3.693704	0.243871	0.048571	26.8	-24	6.9	2.36	50.09653	20.15	0.74
S11	2.216709	0.301323	0.054196	26.8	-24	6.79	2.5	50.68702	28.71	0.82
S12	1.222449	0.198271	0.044355	26.8	-24	6.87	2.3	59.94011	28.69	0.85

TABLE S2. KO (KEGG Orthology) numbers of quorum sensing and cobamide biosynthesis related genes.

Function		KO	code	Description	Reference
**Quorum sensing**	Module 1	K10915	*cqsA*	(S)-3-hydroxytridecan-4-one autoinducer synthase [EC:2.3.-.-]	(Papenfort and Bassler, 2016; Mukherjee and Bassler, 2019; Ng et al., 2009)
		K00060	*tdh*	threonine 3-dehydrogenase [EC:1.1.1.103]	
		K15852	*luxR*	LuxR family transcriptional regulator, transcriptional activator of the bioluminescence operon	
	Module 2	K13061	*rhlI*	acyl homoserine lactone synthase [EC:2.3.1.184]	
		K13060	*lasI*	acyl homoserine lactone synthase [EC:2.3.1.184]	
		K18304	*lasR*	LuxR family transcriptional regulator, quorum-sensing system regulator LasR	
		K17940	*pqsH*	2-heptyl-3-hydroxy-4(1H)-quinolone synthase [EC:1.14.13.182]	
**Cobamide biosynthesis**	Tetrapyrrole precursor biosynthesis	K02492	*hemA*	glutamyl-tRNA reductase [EC:1.2.1.70]	(Warren et al., 2002; Shelton et al., 2019)
		K01698	*hemB*	porphobilinogen synthase [EC:4.2.1.24]	
		K01749	*hemC*	hydroxymethylbilane synthase [EC:2.5.1.61]	
		K02302	*cysG*	uroporphyrin-III C-methyltransferase / precorrin-2 dehydrogenase / sirohydrochlorin ferrochelatase [EC:2.1.1.107 1.3.1.76 4.99.1.4]	
	Anaerobic corrin ring biosynthesis	K03795	*cbiX*	sirohydrochlorin cobaltochelatase [EC:4.99.1.3]	
		K02191	*cbiT*	cobalt-precorrin-6B (C15)-methyltransferase [EC:2.1.1.196]	
		K03399	*cbiE*	cobalt-precorrin-7 (C5)-methyltransferase [EC:2.1.1.289]	
	Anaerobic corrin ring biosynthesis	K02229	*cobG*	precorrin-3B synthase [EC:1.14.13.83]	
		K02228	*cobF*	precorrin-6A synthase [EC:2.1.1.152]	

Adenosylation	K02303	*cobA*	uroporphyrin-III C-methyltransferase [EC:2.1.1.107]
Nucleotide loop assembly	K02227	*cbiB*	adenosylcobinamide-phosphate synthase [EC:6.3.1.10]
	K02225	*cobC*	cobalamin biosynthetic protein CobC
	K09882	*cobS*	cobaltochelatase CobS [EC:6.6.1.2]
Aminopropanol linker	K04720	*cobD*	threonine-phosphate decarboxylase [EC:4.1.1.81]
Alpha-ribazole phosphate biosynthesis	K09883	*cobT*	cobaltochelatase CobT [EC:6.6.1.2]

References

Mukherjee, S., and Bassler, B.L. (2019) Bacterial quorum sensing in complex and dynamically changing environments. *Nat Rev Microbiol* 17(6): 371-382. doi: 10.1038/s41579-019-0186-5

Ng, W.L., and Bassler, B.L. (2009) Bacterial quorum-sensing network architectures. Annu. Rev. Genet. 43, 197-222. doi: 10.1146/annurev-genet-102108-134304

Papenfort, K., and Bassler, B.L. (2016) Quorum sensing signal-response systems in Gram-negative bacteria. *Nat Rev Microbiol* 14(9): 576-588. doi: 10.1038/nrmicro.2016.89

Shelton, A.N., Seth, E.C., Mok, K.C., Han, A.W., Jackson, S.N., Haft, D.R., and Taga, M.E. (2019) Uneven distribution of cobamide biosynthesis and dependence in bacteria predicted by comparative genomics. *ISME J* 13(3): 789-804. doi: 10.1038/s41396-018-0304-9

Warren, M.J., Raux, E., Schubert, H.L., and Escalante-Semerena, J.C. (2002) The biosynthesis of adenosylcobalamin (vitamin B12). *Nat Prod Rep* 19(4): 390-412. doi: 10.1039/B108967F

TABLE S3. Taxonomy information and relative abundance of keystone taxa in bacterial-fungal association (BFA), bacterial and fungal networks across the non-rhizosphere (Non), rhizosphere (Rhi), episphere (Epi) and endosphere (Endo) compartments. Each bacterial operational taxonomic unit (OTU) is represented as OTUB, and each fungal OTU is represented as OTUF.

Network	ID	Compartment	Domain	Phylum	Class	Order	Family	Genus	Species	Abundance
BFA	OTUF_12	Non	Fungi	Basidiomycota	Unclassified	Unclassified	Unclassified	Unclassified	Unclassified	0.00579
BFA	OTUF_34	Non	Fungi	Unclassified	Unclassified	Unclassified	Unclassified	Unclassified	Unclassified	0.00389
BFA	OTUF_87	Non	Fungi	Unclassified	Unclassified	Unclassified	Unclassified	Unclassified	Unclassified	0.00351
BFA	OTUF_101	Non	Fungi	Ascomycota	Unclassified	Unclassified	Unclassified	Unclassified	Unclassified	0.00040
BFA	OTUF_279	Non	Fungi	Unclassified	Unclassified	Unclassified	Unclassified	Unclassified	Unclassified	0.00037
BFA	OTUF_360	Non	Fungi	Ascomycota	Unclassified	Unclassified	Unclassified	Unclassified	Unclassified	0.00223
BFA	OTUF_165	Non	Fungi	Unclassified	Unclassified	Unclassified	Unclassified	Unclassified	Unclassified	0.00110
BFA	OTUF_288	Non	Fungi	Unclassified	Unclassified	Unclassified	Unclassified	Unclassified	Unclassified	0.00052
BFA	OTUF_481	Non	Fungi	Unclassified	Unclassified	Unclassified	Unclassified	Unclassified	Unclassified	0.00040
BFA	OTUF_8338	Non	Fungi	Unclassified	Unclassified	Unclassified	Unclassified	Unclassified	Unclassified	0.00063
BFA	OTUB_153	Non	Bacteria	Chloroflexi	Anaerolineae	SBR1031	*A4b*	Unclassified		0.00098
BFA	OTUB_2165	Non	Bacteria	Proteobacteria	Gammaproteobacteria	Alteromonadales	*OM60*	*Congregibacter*		0.00026
BFA	OTUB_369	Non	Bacteria	Acidobacteria	Acidobacteria-6	CCU21	Unclassified	Unclassified		0.00109
BFA	OTUB_37	Non	Bacteria	Chloroflexi	Anaerolineae	GCA004	Unclassified	Unclassified		0.00218
BFA	OTUB_659	Non	Bacteria	Chloroflexi	Dehalococcoidetes	Dehalococcoidales	*Dehalococcoidaceae*	Unclassified		0.00018
BFA	OTUF_175	Rhi	Fungi	Ascomycota	Unclassified	Unclassified	Unclassified	Unclassified	Unclassified	0.00052
BFA	OTUF_327	Rhi	Fungi	Unclassified	Unclassified	Unclassified	Unclassified	Unclassified	Unclassified	0.00024
BFA	OTUF_552	Rhi	Fungi	Unclassified	Unclassified	Unclassified	Unclassified	Unclassified	Unclassified	0.00029
BFA	OTUF_689	Rhi	Fungi	Unclassified	Unclassified	Unclassified	Unclassified	Unclassified	Unclassified	0.00014

BFA	OTUF_703	Rhi	Fungi	Unclassified	Unclassified	Unclassified	Unclassified	Unclassified	Unclassified	0.00028
BFA	OTUF_790	Rhi	Fungi	Unclassified	Unclassified	Unclassified	Unclassified	Unclassified	Unclassified	0.00016
BFA	OTUF_1192	Rhi	Fungi	Unclassified	Unclassified	Unclassified	Unclassified	Unclassified	Unclassified	0.00023
BFA	OTUF_2514	Rhi	Fungi	Basidiomycota	Agaricomycetes	Unclassified	Unclassified	Unclassified	Unclassified	0.00011
BFA	OTUF_33	Rhi	Fungi	Unclassified	Unclassified	Unclassified	Unclassified	Unclassified	Unclassified	0.00117
BFA	OTUF_658	Rhi	Fungi	Ascomycota	Unclassified	Unclassified	Unclassified	Unclassified	Unclassified	0.00028
BFA	OTUF_667	Rhi	Fungi	Basidiomycota	Agaricomycetes	Unclassified	Unclassified	Unclassified	Unclassified	0.00013
BFA	OTUF_764	Rhi	Fungi	Ascomycota	Unclassified	Unclassified	Unclassified	Unclassified	Unclassified	0.00029
BFA	OTUF_2579	Rhi	Fungi	Unclassified	Unclassified	Unclassified	Unclassified	Unclassified	Unclassified	0.00030
BFA	OTUF_15613	Rhi	Fungi	Unclassified	Unclassified	Unclassified	Unclassified	Unclassified	Unclassified	0.00037
BFA	OTUB_3155	Rhi	Bacteria	Proteobacteria	Gammaproteobacteria	Alteromonadales	*OM60*	Unclassified		0.00020
BFA	OTUB_339	Rhi	bacteria	Proteobacteria	Alphaproteobacteria	Rhizobiales	*Hyphomicrobiaceae*	*Rhodoplanes*		0.00099
BFA	OTUB_1295	Rhi	Bacteria	Proteobacteria	Deltaproteobacteria	Desulfobacterales	*Desulfobulbaceae*	Unclassified		0.00021
BFA	OTUB_18	Rhi	Bacteria	Proteobacteria	Deltaproteobacteria	Syntrophobacterales	*Syntrophobacteraceae*	Unclassified		0.00193
BFA	OTUB_494	Rhi	Bacteria	Cyanobacteria	Chloroplast	Stramenopiles	Unclassified	Unclassified		0.00035
BFA	OTUB_615	Rhi	Bacteria	Proteobacteria	Deltaproteobacteria	Desulfobacterales	*Desulfobulbaceae*	Unclassified		0.0014
BFA	OTUB_756	Rhi	Bacteria	Proteobacteria	Deltaproteobacteria	Myxococcales	Unclassified	Unclassified		0.00058
BFA	OTUB_943	Rhi	Bacteria	Gemmatimonadetes	Gemm-2	Unclassified	Unclassified	Unclassified		0.0020
BFA	OTUB_995	Rhi	Bacteria	Caldithrix	Caldithrixae	Caldithrixales	*Caldithrixaceae*	*LCP-26*		0.0001
BFA	OTUF_250	Endo	Fungi	Unclassified	Unclassified	Unclassified	Unclassified	Unclassified	Unclassified	0.00024
BFA	OTUF_361	Endo	Fungi	Unclassified	Unclassified	Unclassified	Unclassified	Unclassified	Unclassified	0.00032
BFA	OTUF_48	Endo	Fungi	Unclassified	Unclassified	Unclassified	Unclassified	Unclassified	Unclassified	0.00037
BFA	OTUF_155	Endo	Fungi	Unclassified	Unclassified	Unclassified	Unclassified	Unclassified	Unclassified	0.00008
BFA	OTUF_163	Endo	Fungi	Ascomycota	Unclassified	Unclassified	Unclassified	Unclassified	Unclassified	0.00024
BFA	OTUF_407	Endo	Fungi	Unclassified	Unclassified	Unclassified	Unclassified	Unclassified	Unclassified	0.00023
BFA	OTUF_1515	Endo	Fungi	Ascomycota	Unclassified	Unclassified	Unclassified	Unclassified	Unclassified	0.00013
BFA	OTUB_260	Endo	Bacteria	Proteobacteria	Deltaproteobacteria	Desulfarculales	*Desulfarculaceae*	Unclassified		0.00088
BFA	OTUB_472	Endo	Bacteria	Chloroflexi	Anaerolineae	Unclassified	Unclassified	Unclassified		0.00016

BFA	OTUB_120	Endo	Bacteria	Proteobacteria	Alphaproteobacteria	Rhizobiales	*Rhizobiaceae*	Unclassified	0.00014
BFA	OTUB_1491	Endo	Bacteria	Proteobacteria	Gammaproteobacteria	Vibrionales	*Vibrionaceae*	*Vibrio*	0.00024
BFA	OTUB_2009	Endo	Bacteria	Chloroflexi	Anaerolineae	S0208	Unclassified	Unclassified	0.00004
BFA	OTUB_304	Endo	Bacteria	Proteobacteria	Deltaproteobacteria	Desulfobacterales	*Desulfobacteraceae*	*Desulfococcus*	0.00026
Bacterial	OTUB_32	Non	Bacteria	Proteobacteria	Deltaproteobacteria	Desulfobacterales	*Desulfobulbaceae*	Unclassified	0.003213
Bacterial	OTUB_38	Non	Bacteria	Cyanobacteria	Chloroplast	Stramenopiles	Unclassified	Unclassified	0.010592
Bacterial	OTUB_1072	Non	Bacteria	Chloroflexi	Anaerolineae	GCA004	Unclassified	Unclassified	0.000147
Bacterial	OTUB_216	Non	Bacteria	Proteobacteria	Gammaproteobacteria	[Marinicellales]	*[Marinicellaceae]*	Unclassified	7.73E-04
Bacterial	OTUB_224	Non	Bacteria	Firmicutes	Clostridia	Clostridiales	*Clostridiaceae*	Clostridium	0.000299
Bacterial	OTUB_607	Non	Bacteria	Planctomycetes	OM190	CL500-15	Unclassified	Unclassified	0.000225
Bacterial	OTUB_686	Non	Bacteria	Acidobacteria	OS-K	Unclassified	Unclassified	Unclassified	5.06E-04
Bacterial	OTUB_98	Non	Bacteria	Nitrospirae	Nitrospira	Nitrospirales	*[Thermodesulfovibrionaceae]*	*LCP-6*	0.001022
Bacterial	OTUB_185	Rhi	Bacteria	Chloroflexi	Anaerolineae	S0208	Unclassified	Unclassified	0.002146
Bacterial	OTUB_267	Rhi	Bacteria	Proteobacteria	Alphaproteobacteria	Rhodospirillales	Unclassified	Unclassified	0.00045
Bacterial	OTUB_369	Rhi	Bacteria	Acidobacteria	Acidobacteria-6	CCU21	Unclassified	Unclassified	0.002521
Bacterial	OTUB_462	Rhi	Bacteria	Proteobacteria	Deltaproteobacteria	Desulfuromonadales	*Desulfuromonadaceae*	*Pelobacter*	0.000645
Bacterial	OTUB_469	Rhi	Bacteria	Proteobacteria	Alphaproteobacteria	Rhodobacterales	*Rhodobacteraceae*	*Amaricoccus*	0.000682
Bacterial	OTUB_527	Rhi	Bacteria	Chloroflexi	Anaerolineae	GCA004	Unclassified	Unclassified	0.000757
Bacterial	OTUB_68	Rhi	Bacteria	Proteobacteria	Alphaproteobacteria	Rhizobiales	*Hyphomicrobiaceae*	Unclassified	0.002398
Bacterial	OTUB_736	Rhi	Bacteria	Proteobacteria	Gammaproteobacteria	Thiotrichales	*Piscirickettsiaceae*	Unclassified	0.000613
Bacterial	OTUB_1144	Rhi	Bacteria	Proteobacteria	Deltaproteobacteria	Desulfobacterales	*Desulfobulbaceae*	Unclassified	0.00018
Bacterial	OTUB_1224	Rhi	Bacteria	Proteobacteria	Gammaproteobacteria	HTCC2188	*HTCC2089*	Unclassified	0.000221
Bacterial	OTUB_200	Rhi	Bacteria	Proteobacteria	Deltaproteobacteria	Desulfobacterales	*Desulfobacteraceae*	Unclassified	0.000728
Bacterial	OTUB_2433	Rhi	Bacteria	Gemmatimonadetes	Gemm-2	Unclassified	Unclassified	Unclassified	0.000227
Bacterial	OTUB_276	Rhi	Bacteria	Proteobacteria	Alphaproteobacteria	Rhizobiales	*Hyphomicrobiaceae*	Unclassified	0.000784
Bacterial	OTUB_295	Rhi	Bacteria	Caldithrix	Caldithrixae	Caldithrixales	*Caldithrixaceae*	*LCP-26*	0.000963
Bacterial	OTUB_306	Rhi	Bacteria	Chloroflexi	Anaerolineae	GCA004	Unclassified	Unclassified	0.000506

Bacterial	OTUB_3076	Rhi	Bacteria	Proteobacteria	Alphaproteobacteria	Unclassified	Unclassified	Unclassified		0.000532
Bacterial	OTUB_516	Rhi	Bacteria	Caldithrix	Caldithrixae	Caldithrixales	*Caldithrixaceae*	*LCP-26*		0.000408
Bacterial	OTUB_862	Rhi	Bacteria	Proteobacteria	Alphaproteobacteria	Sphingomonadales	*Sphingomonadaceae*	Unclassified		0.000287
Bacterial	OTUB_891	Rhi	Bacteria	Proteobacteria	Alphaproteobacteria	Rhodospirillales	Unclassified	Unclassified		0.000491
Bacterial	OTUB_943	Rhi	Bacteria	Gemmatimonadetes	Gemm-2	Unclassified	Unclassified	Unclassified		0.000429
Bacterial	OTUB_100	Epi	Bacteria	Chloroflexi	Anaerolineae	SBR1031	*SHA-31*	Unclassified		0.001628
Bacterial	OTUB_110	Epi	Bacteria	Chloroflexi	Ellin6529	Unclassified	Unclassified	Unclassified		0.001139
Bacterial	OTUB_1267	Epi	Bacteria	Proteobacteria	Alphaproteobacteria	Rhodospirillales	*Rhodospirillaceae*	Unclassified		0.000283
Bacterial	OTUB_3507	Epi	Bacteria	Chloroflexi	Anaerolineae	SBR1031	SHA-31	Unclassified		0.001632
Bacterial	OTUB_390	Epi	Bacteria	Proteobacteria	Deltaproteobacteria	[Entotheonellales]	Unclassified	Unclassified		0.000893
Bacterial	OTUB_405	Epi	Bacteria	WS2	SHA-109	Unclassified	Unclassified	Unclassified		0.000382
Bacterial	OTUB_480	Epi	Bacteria	Actinobacteria	OPB41	Unclassified	Unclassified	Unclassified		0.000356
Bacterial	OTUB_1160	Epi	Bacteria	Proteobacteria	Deltaproteobacteria	Desulfobacterales	*Desulfobulbaceae*	Unclassified		0.000537
Bacterial	OTUB_164	Epi	Bacteria	OD1	ZB2	Unclassified	Unclassified	Unclassified		0.000865
Bacterial	OTUB_1643	Epi	Bacteria	Bacteroidetes	[Rhodothermi]	[Rhodothermales]	*Rhodothermaceae*	Unclassified		0.000313
Bacterial	OTUB_1682	Epi	Bacteria	Chloroflexi	SAR202	Unclassified	Unclassified	Unclassified		0.000312
Bacterial	OTUB_183	Epi	Bacteria	Chloroflexi	Anaerolineae	SBR1031	*SJA-101*	Unclassified		0.000322
Bacterial	OTUB_431	Epi	Bacteria	Gemmatimonadetes	Gemm-2	Unclassified	Unclassified	Unclassified		0.000249
Bacterial	OTUB_4411	Epi	Bacteria	Proteobacteria	Alphaproteobacteria	Rhizobiales	*Methylocystaceae*	*Pleomorphomonas*		0.000237
Bacterial	OTUB_120	Endo	Bacteria	Proteobacteria	Alphaproteobacteria	Rhizobiales	*Rhizobiaceae*	Unclassified		0.000314
Bacterial	OTUB_260	Endo	Bacteria	Proteobacteria	Deltaproteobacteria	Desulfarculales	*Desulfarculaceae*	Unclassified		0.001976
Bacterial	OTUB_4837	Endo	Bacteria	Proteobacteria	Gammaproteobacteria	Oceanospirillales	*Oceanospirillaceae*	*Marinomonas*		8.83E-05
Bacterial	OTUB_877	Endo	Bacteria	Proteobacteria	Deltaproteobacteria	Myxococcales	Unclassified	Unclassified		0.000652
Bacterial	OTUB_150	Endo	Bacteria	Chloroflexi	Anaerolineae	CFB-26	Unclassified	Unclassified		0.000474
Fungal	OTUF_1190	Non	Fungi	Basidiomycota	Unclassified	Unclassified	Unclassified	Unclassified	Unclassified	3.23E-04
Fungal	OTUF_3052	Non	Fungi	Unclassified	Unclassified	Unclassified	Unclassified	Unclassified	Unclassified	0.000585
Fungal	OTUF_360	Non	Fungi	Ascomycota	Unclassified	Unclassified	Unclassified	Unclassified	Unclassified	4.14E-03

Fungal	OTUF_647	Non	Fungi	Ascomycota	Unclassified	Unclassified	Unclassified	Unclassified	Unclassified	0.000214
Fungal	OTUF_133	Non	Fungi	Basidiomycota	Unclassified	Unclassified	Unclassified	Unclassified	Unclassified	2.04E-03
Fungal	OTUF_14596	Non	Fungi	Unclassified	Unclassified	Unclassified	Unclassified	Unclassified	Unclassified	2.47E-04
Fungal	OTUF_217	Non	Fungi	Basidiomycota	Unclassified	Unclassified	Unclassified	Unclassified	Unclassified	3.65E-04
Fungal	OTUF_256	Non	Fungi	Unclassified	Unclassified	Unclassified	Unclassified	Unclassified	Unclassified	0.001048
Fungal	OTUF_307	Non	Fungi	Ascomycota	Unclassified	Unclassified	Unclassified	Unclassified	Unclassified	0.000697
Fungal	OTUF_3436	Non	Fungi	Ascomycota	Unclassified	Unclassified	Unclassified	Unclassified	Unclassified	2.32E-04
Fungal	OTUF_447	Non	Fungi	Ascomycota	Unclassified	Unclassified	Unclassified	Unclassified	Unclassified	1.79E-03
Fungal	OTUF_175	Rhi	Fungi	Ascomycota	Unclassified	Unclassified	Unclassified	Unclassified	Unclassified	0.000959
Fungal	OTUF_29	Rhi	Fungi	Unclassified	Unclassified	Unclassified	Unclassified	Unclassified	Unclassified	0.003804
Fungal	OTUF_15613	Rhi	Fungi	Unclassified	Unclassified	Unclassified	Unclassified	Unclassified	Unclassified	0.000682
Fungal	OTUF_21	Rhi	Fungi	Ascomycota	Unclassified	Unclassified	Unclassified	Unclassified	Unclassified	0.006385
Fungal	OTUF_2251	Rhi	Fungi	Unclassified	Unclassified	Unclassified	Unclassified	Unclassified	Unclassified	0.000488
Fungal	OTUF_238	Rhi	Fungi	Unclassified	Unclassified	Unclassified	Unclassified	Unclassified	Unclassified	0.000189
Fungal	OTUF_658	Rhi	Fungi	Ascomycota	Unclassified	Unclassified	Unclassified	Unclassified	Unclassified	0.000511
Fungal	OTUF_668	Rhi	Fungi	Ascomycota	Unclassified	Unclassified	Unclassified	Unclassified	Unclassified	0.000952
Fungal	OTUF_703	Rhi	Fungi	Unclassified	Unclassified	Unclassified	Unclassified	Unclassified	Unclassified	0.000527
Fungal	OTUF_764	Rhi	Fungi	Ascomycota	Unclassified	Unclassified	Unclassified	Unclassified	Unclassified	0.000548
Fungal	OTUF_82	Rhi	Fungi	Unclassified	Unclassified	Unclassified	Unclassified	Unclassified	Unclassified	0.001569
Fungal	OTUF_832	Rhi	Fungi	Ascomycota	Unclassified	Unclassified	Unclassified	Unclassified	Unclassified	0.000385
Fungal	OTUF_867	Rhi	Fungi	Unclassified	Unclassified	Unclassified	Unclassified	Unclassified	Unclassified	0.000489
Fungal	OTUF_106	Epi	Fungi	Basidiomycota	Unclassified	Unclassified	Unclassified	Unclassified	Unclassified	0.001713
Fungal	OTUF_1730	Epi	Fungi	Ascomycota	Unclassified	Unclassified	Unclassified	Unclassified	Unclassified	0.000892
Fungal	OTUF_237	Epi	Fungi	Ascomycota	Unclassified	Unclassified	Unclassified	Unclassified	Unclassified	0.000652
Fungal	OTUF_3612	Epi	Fungi	Unclassified	Unclassified	Unclassified	Unclassified	Unclassified	Unclassified	0.001599
Fungal	OTUF_4100	Epi	Fungi	Unclassified	Unclassified	Unclassified	Unclassified	Unclassified	Unclassified	0.000227
Fungal	OTUF_634	Epi	Fungi	Basidiomycota	Unclassified	Unclassified	Unclassified	Unclassified	Unclassified	0.000181
Fungal	OTUF_82	Epi	Fungi	Unclassified	Unclassified	Unclassified	Unclassified	Unclassified	Unclassified	0.003523

Fungal	OTUF_119	Epi	Fungi	Unclassified	Unclassified	Unclassified	Unclassified	Unclassified	Unclassified	0.001653
Fungal	OTUF_1440	Epi	Fungi	Unclassified	Unclassified	Unclassified	Unclassified	Unclassified	Unclassified	0.001319
Fungal	OTUF_154	Epi	Fungi	Ascomycota	Sordariomycetes	Unclassified	Unclassified	Unclassified	Unclassified	0.001383
Fungal	OTUF_2514	Epi	Fungi	Basidiomycota	Agaricomycetes	Unclassified	Unclassified	Unclassified	Unclassified	0.000356
Fungal	OTUF_4527	Epi	Fungi	Ascomycota	Unclassified	Unclassified	Unclassified	Unclassified	Unclassified	0.000292
Fungal	OTUF_581	Epi	Fungi	Basidiomycota	Unclassified	Unclassified	Unclassified	Unclassified	Unclassified	0.002062
Fungal	OTUF_220	Endo	Fungi	Basidiomycota	Unclassified	Unclassified	Unclassified	Unclassified	Unclassified	0.000689
Fungal	OTUF_3013	Endo	Fungi	Basidiomycota	Unclassified	Unclassified	Unclassified	Unclassified	Unclassified	0.000586
Fungal	OTUF_34	Endo	Fungi	Unclassified	Unclassified	Unclassified	Unclassified	Unclassified	Unclassified	7.98E-05
Fungal	OTUF_7507	Endo	Fungi	Unclassified	Unclassified	Unclassified	Unclassified	Unclassified	Unclassified	0.00086

TABLE S4. Key network topological characteristics for bacterial, fungal and bacterial-fungal association (BFA) networks across the non-rhizosphere (Non), rhizosphere (Rhi), episphere (Epi) and endosphere (Endo) compartments.

Compartment	Bacteria networks			Fungal networks			BFA networks		
	R square of power-law	Average connectivity	Modularity values	R square of power-law	Average connectivity	Modularity values	R square of power-law	Average connectivity	Modularity values
Non	0.914	3.681	0.710	0.874	4.921	0.707	0.908	2.834	0.851
Rhi	0.911	5.039	0.615	0.888	7.296	0.522	0.920	4.144	0.678
Epi	0.915	3.686	0.727	0.883	3.799	0.756	0.929	1.731	0.913
Endo	0.905	2.628	0.754	0.921	2.419	0.751	0.860	2.986	0.796

TABLE S5. Root exudates that related to biofilm formation and cobamide biosynthesis. EPS represented extracellular polymeric substances, N represented negative mode, and P represented positive mode.

Metabolism	Compound	Median retention time (Second)	Function	Reference
Biofilm formation	Trehalose (EPS)	147.22 (N)<br>149.87 (P)	Stimulate the formation of biofilms on fungal hyphae	(Karygianni et al., 2020)
	Glucan (EPS)	48.13 (N)<br>345.06 (P)	Adhesion, cohesion, scaffolding, stability, cell-to-cell binding, acidic microenvironment, protection against antimicrobials, nutrient	
Cobamide biosynthesis	Adenine	107.68 (N)<br>149.88 (P)	Participating in the biosynthesis of lower ligand structure of cobamide	(Shelton et al., 2019)

Reference

Karygianni, L., Ren, Z., Koo, H., and Thurnheer, T. (2020). Biofilm Matrixome, Extracellular Components in Structured Microbial Communities. Trends Microbiol. 28, 668-681. doi: 10.1016/j.tim.2020.03.016

Shelton, A.N., Seth, E.C., Mok, K.C., Han, A.W., Jackson, S.N., and Haft, D.R. (2019). Uneven distribution of cobamide biosynthesis and dependence in bacteria predicted by comparative genomics. ISME J. 13, 789-804. doi: 10.1038/s41396-018-0304-9
